# Thysanoptera-Terebrantia of the Hawaiian Islands: an identification manual

**DOI:** 10.3897/zookeys.549.6889

**Published:** 2016-01-05

**Authors:** Laurence Mound, Sueo Nakahara, Dick M. Tsuda

**Affiliations:** 1 Australian National Insect Collection, CSIRO, Canberra, Australia; 2 retired from Systematic Entomology Laboratory, USDA-ARS, Beltsville, Maryland, USA; 3 Department of Entomology, University of Hawaii, Honolulu, Hawaii, USA

**Keywords:** Thysanoptera, Terebrantia, identification keys, Hawaiian Islands, endemics, adventives

## Abstract

An illustrated identification system is presented to 99 species and 49 genera in three families recorded from the Hawaiian Islands in the Thysanoptera suborder Terebrantia. Only seven (possibly eight) of these species are considered endemic, the remainder being adventive to these islands. The only previous study of Hawaiian Thysanoptera, by Zimmerman in 1948, included 47 Terebrantia species in 21 genera.

## Preamble

This paper derives from a 200 page un-illustrated typescript prepared by Steve Nakahara some years prior to his retirement in 1998 from work on thrips. His co-author, Dick Tsuda, sent that typescript to Laurence Mound in June 2015, and in the absence of funding to complete such an extensive work, this annotated identification manual was prepared in Canberra, Australia. Records of taxa from particular Hawaiian Islands are copied from the original typescript.

## Introduction

This account of the Thysanoptera-Terebrantia known from the Hawaiian Islands includes 99 species, of which only seven (or possibly eight) are considered endemic and over 90 species are considered adventive from other parts of the world. The obvious conclusion from these numbers is, *either* the native thrips fauna of Hawaii is particularly small, *or* collecting activity for thrips associated with the native flora is inadequate. The data provided here thus contribute little to our understanding of island biogeography, but emphasise two matters of considerable importance to plant quarantine and plant protection: 1, that thrips are particularly prone to dispersal through commercial activities, especially the horticultural trade in live plants which harbor undetected thrips contaminants, and 2, that Hawaii is particularly susceptible to such invaders because almost half of the 1800 established flowering plant species are non-native (Wagner et al. 1990). The only previous account of Hawaiian thrips was provided by Zimmerman (1948). He reviewed earlier studies, but used a classification that is now seriously outdated. Amongst the suborder Terebrantia from Hawaii Zimmerman listed only 47 species in 21 genera and two families. Of those 47 species, 30 have since undergone some sort of nomenclatural change, and subsequent workers, particularly Nakahara, Sakimura and Tsuda, have recorded many additional taxa (see Appendix). As a result, Nishida et al. (1992) listed 83 species in 44 genera of Terebrantia, although Bernarr Kumashiro kindly provided (August 2015) an unpublished manuscript listing a further seven species identified by Sueo Nakahara. The Bishop Museum Hawaiian Arthropod Checklist (2015) includes 92 Terebrantia names. However, that list is both incomplete and includes several unsubstantiated records, and moreover nomenclature of some taxa is out of date.

General information about Thysanoptera including identification systems are available on several web-sites, including Hoddle et al. (2012) and Mound et al. (2012), and full nomenclatural information about these insects is available in ThripsWiki (2015). General information about thrips in the form of hard-copy is available in Mound and Marullo (1996), and also Stannard (1968), although the nomenclature in the latter is seriously out-of-date. The Appendix given below, following Literature Cited, lists many published reports by authors in Hawaii on the thrips fauna of these islands.

## Biology of Thysanoptera

About 50% of Thysanoptera species feed only on fungi that develop on dead leaves, twigs and branches of woody higher plants (Morse and Hoddle 2006). These fungivorous thrips are all members of the single family in the suborder Tubulifera, and as such are not considered here. The species discussed in this presentation, the Terebrantia, feed on the tissues of living plants, on leaves, flowers and fruit surfaces, with a few species predatory on other small arthropods. It is members of this suborder that so often cause feeding damage to agricultural and horticultural crops, with several species transmitting damaging tospoviruses.

An important aspect of the biology of thrips is the identity of the plants on which individual species can maintain populations, although this relationship can be difficult to establish (Mound 2013). Adults of many species are highly dispersive, and land on plants on which they cannot breed or even feed. However, in published literature the names of plants from which adult thrips have been collected are commonly quoted as “hosts”, depite the lack of evidence of feeding let alone breeding on such plants. Thrips species of some genera exhibit considerable levels of host specificity. Many genera are associated only with Poaceae, the grasses and bamboos (Mound 2011b). *Dichromothrips* species are specific to Orchidaceae, and *Projectothrips* specific to *Pandanus* flowers. But the larger genera such as *Frankliniella*, *Scirtothrips* and *Thrips* include many species that appear to be generalists, but with other species that are host specific. One important aspect of the generalist species is that 13 of them are currently recognized as the vectors of various Tospovirus species that cause damage to many crops (Riley et al. 2011).

### Thysanoptera as migrants

Given that the only recorded endemic Hawaiian Terebrantia are seven described species of *Neurisothrips*, and possibly one species of *Projectothrips*, the origin of the remaining 90 species is of considerable plant protection interest. At least 50 of these species are found widely around the world. For these, the country in which they originally evolved is probably of limited importance when considering invasion pathways. Such species could have reached Hawaii from any part of their disrupted distribution, although Yamanaka et al. (2015) appeared to assume that each invasive species had entered Hawaii from the area in which it had evolved. Despite this, an invasion pathway from the Americas is indicated by the 15 species that are found otherwise only in North America, and a second pathway is indicated by the five species shared only with the Neotropics. Similarly 15 species are known only from Southeast Asia or Australia, and a trans-Pacific pathway is presumably responsible for their introduction into Hawaii.

## Taxonomy of Thysanoptera

The members of this Order of insects are known as thrips. This word is a plural noun, such that whether “two thrips” or just “one thrips”, the word thrips remains unchanged in the same way that the word sheep is both singular and plural. The word thrips is a Greek word for woodworm, because many of the early records of these insects referred to fungus-feeding species that live on dead branches. Thrips are slender, elongate insects, usually flattened dorso-ventrally, and the adults range from 0.5 to 15.0 mm in length. Adults and larvae are unusual amongst insects in having only a single mandible, the left one, because the right mandible is resorbed early in development. The other character state of thrips that is almost unique amongst adult insects is the absence of tarsal claws and the presence of a tarsal arolium. In contrast, the characteristic fringed wings from which the ordinal name is derived occur in many other small insects, including beetles, wasps, moths and caddisflies. However, adults of many thrips species are apterous or micropterous – that is, wingless or with very short wings.

Two suborders are recognised, the Terebrantia and the Tubulifera. Only one family is recognised in the Tubulifera, but eight families are recognised in the Terebrantia (plus a further five families for fossil taxa) (ThripsWiki 2015).

### Technical terms

The following technical terms are used in the keys provided here. In association with the three ocelli on the head there are commonly three pairs of setae: ocellar setal pair I in front of the first ocellus, pair II arise laterally close to the compound eyes, pair III vary in position from inside to just outside the ocellar triangle. Ocellar setal pair I are absent in species of *Taeniothrips* and *Thrips* (Figs 107, 111), but present in species of *Frankliniella* (Figs 65–67). When referring to setae on the tergites and sternites, it is usual to consider the pair nearest the body mid-line as setal pair S1, the other setae then being numbered sequentially away from the mid-line. The abdominal tergites of species in *Frankliniella* and *Thrips* and related genera bear laterally a pair of organized microtrichial rows referred to as ctenidia (Figs 14, 15). The tergites, and/or the sternites, sometimes bear a posteromarginal flange or craspedum, and this may be entire or lobed in various ways (Figs 38, 47). Campaniform sensilla are pore-like structures (Figs 14, 27) that are presumably stretch receptors on the chitinous surface of various parts of the thorax and abdomen.

**Figures 1–15.**
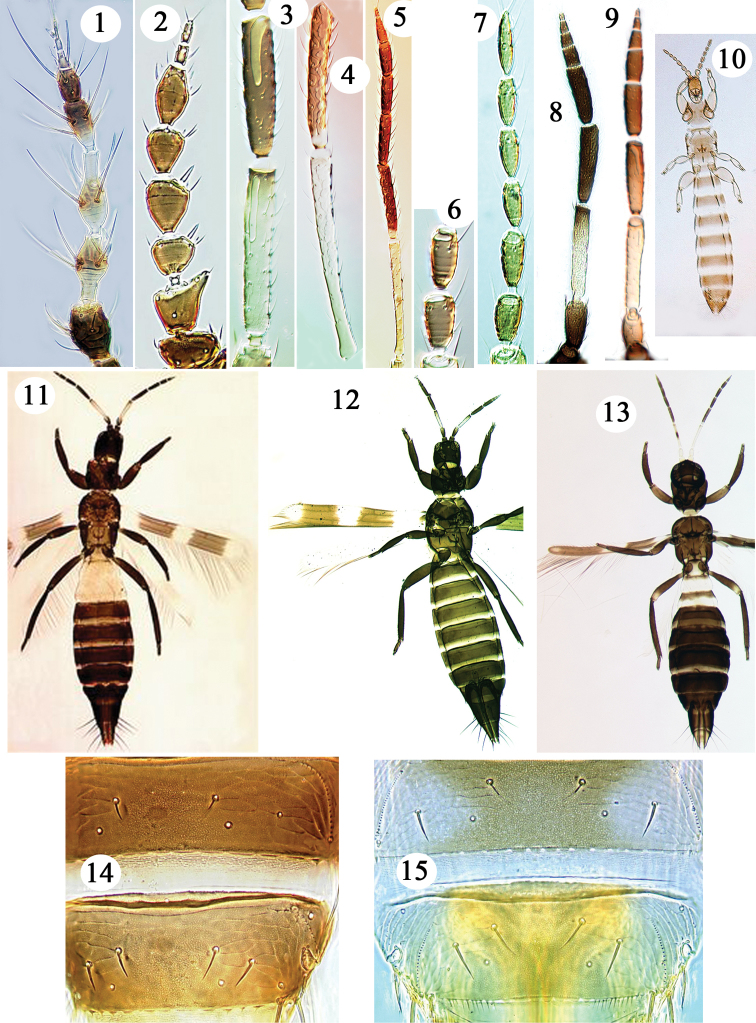
Terebrantia from Hawaii. Antennae **1–9**: **1** *Selenothrips rubrocinctus* **2** *Chirothrips manicatus* **3** *Aeolothrips fasciatus* segments III–IV **4** *Franklinothrips vespiformis* segments III–IV **5** *Franklinothrips vespiformis* **6** *Merothrips floridensis* segments III–IV **7** *Merothrips floridensis* **8** *Aeolothrips fasciatus* **9** *Aeolothrips nasturtii*. Females **10–13**: **10** *Merothrips morgani* **11** *Aeolothrips bicolor* **12** *Aeolothrips fasciatus* **13** *Franklinothrips vespiformis*. Tergites VII & VIII 14–15: **14** *Frankliniella schultzei* **15** *Thrips australis*.

**Figures 16–28.**
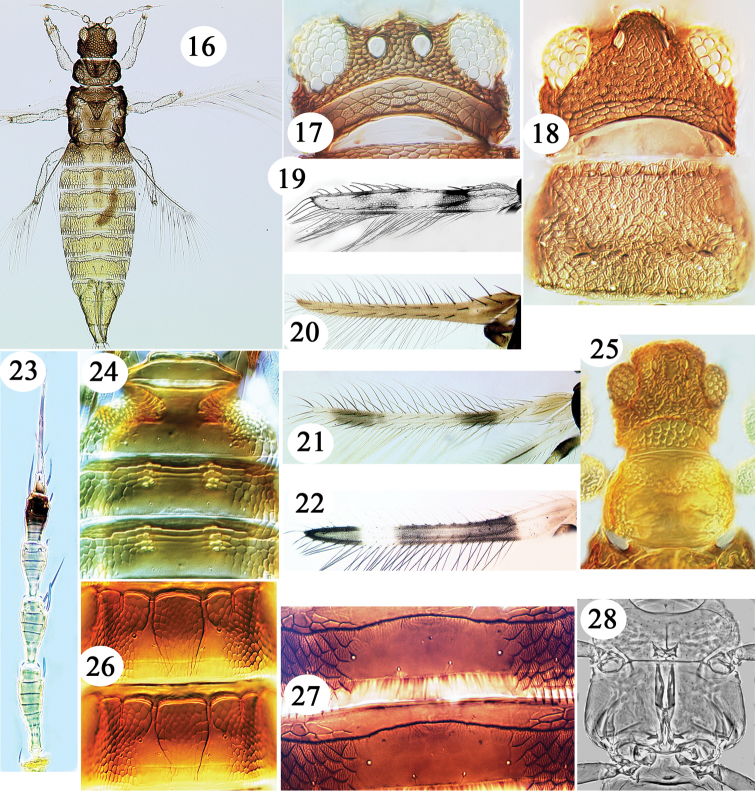
Thripidae from Hawaii. **16** *Heliothrips haemorroidalis*. **17** *Helionothrips errans*, head **18** *Caliothrips fasciatus*, head & pronotum. Fore wing **19–22**: **19** *Parthenothrips dracaenae* **20** *Selenothrips rubrocinctus* **21** *Hercinothrips bicinctus* **22** *Caliothrips fasciatus*. **23** *Heliothrips haemorroidalis* antenna **24** *Anisopilothrips venustulus*, tergites I–III. Tergites III–IV **26–27**: **26** *Helionothrips errans* **27** *Caliothrips fasciatus*. **28** *Asprothrips seminigricornis* metathoracic furca.

**Figures 29–44.**
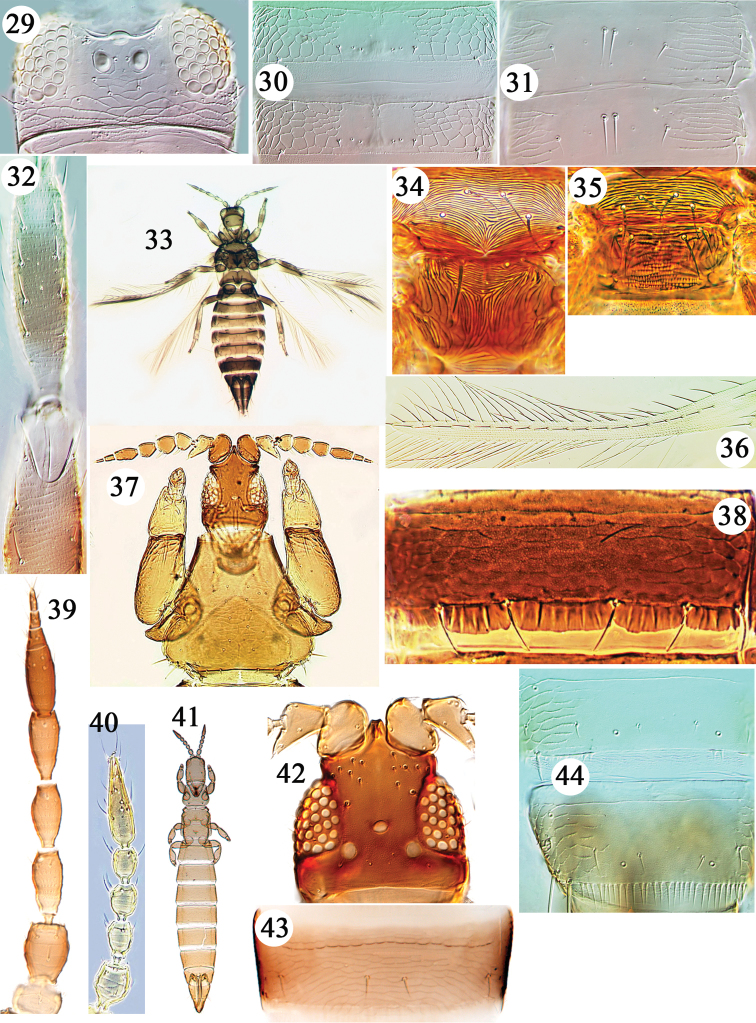
Thripidae from Hawaii. *Asprothrips seminigricornis* **29–30**: **29** head **30** tergites III & IV **31** *Leucothrips theobromae*, tergites III & IV. *Neohydatothrips samayunkur* **32–34**: **32** fore tibia **33** female **34** meso & metanotum. **35** *Sericothrips staphylinus* meso & metanotum. **36** *Neohydatothrips gracilipes* fore wing **37** *Arorathrips mexicanus*, head & pronotum **38** *Apterothrips apteris* sternite IV **39** *Anaphothrips obscurus* antenna. *Aptinothrips rufus* **40–41**: **40** antenna **41** female **42** *Arorathrips spiniceps*, head **43** *Arorathrips mexicanus* tergite IV **44** *Anaphothrips swezeyi* tergites VII–VIII.

**Figures 45–64.**
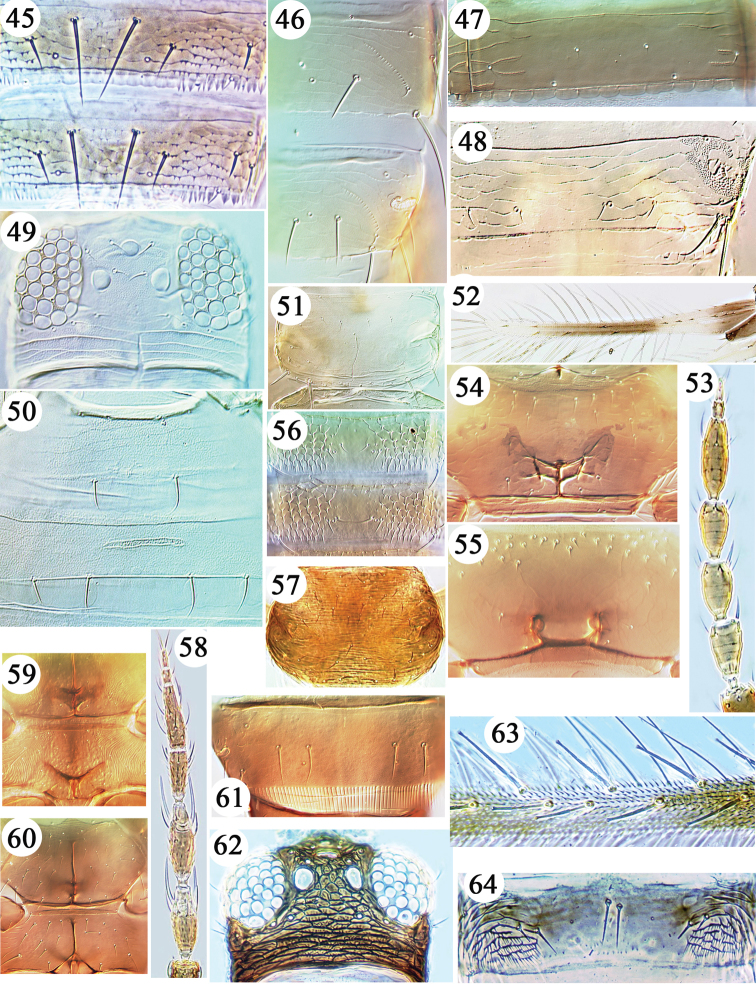
Thripinae from Hawaii. **45** *Baileyothrips arizonensis* tergites IV & V. **46** *Bolacothrips striatopterus* tergites VII & VIII. **47** *Bregmatothrips venustus* tergite IV. **48** *Chaetanaphothrips leeuweni* tergite VIII. *Chaetanaphothrips signipennis* **49–50**: **49** head **50** sternites II & III. *Chaetanaphothrips orchidii* **51–52**: **51** pronotum **52** fore wing **53** *Limothrips cerealium* antenna. Metathoracic furca **54–55**: **54** *Chirothrips manicatus* **55** *Arorathrips mexicanus* **56** *Dendrothripoides innoxius* tergites. *Dichromothrips corbetti* **57–59**: **57** pronotum **58** antenna **59** meso & metathoracic furcae. *Dichromothrips smithi* **60–61**: **60** meso & metathoracic furcae **61** tergite VIII. *Echinothrips americanus* **62–64**: **62** head **63** fore wing setae **64** tergite IV.

**Figures 65–82.**
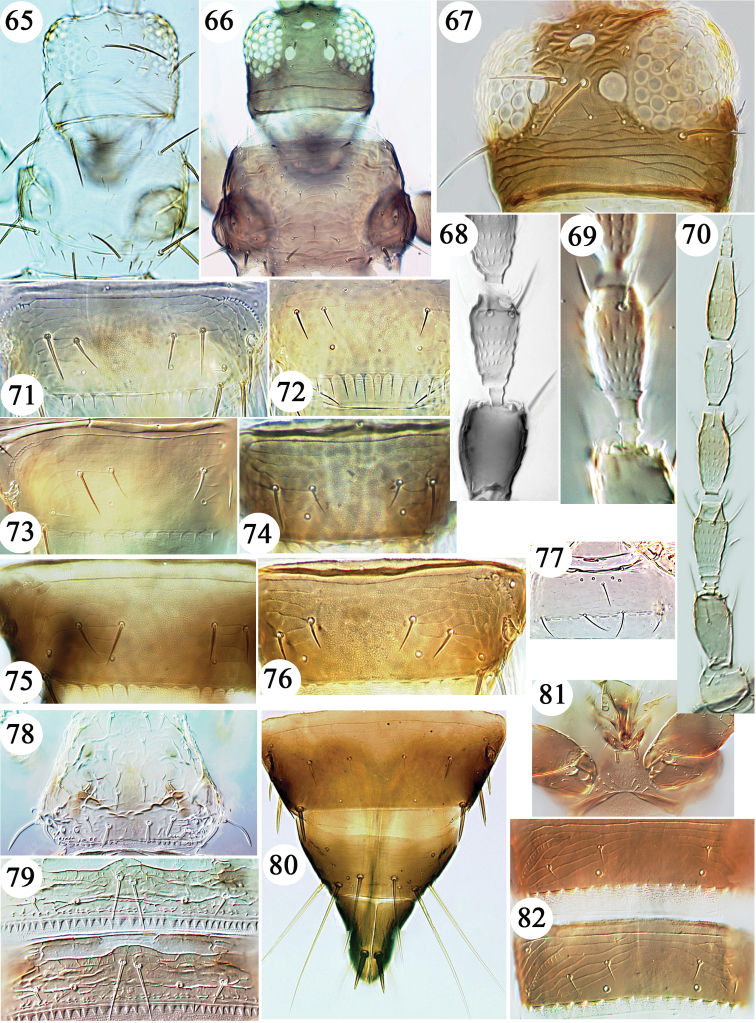
Thripinae from Hawaii. **65** *Frankliniella occidentalis* head & pronotum. **66** *Frankliniella minuta* head & pronotum **67** *Frankliniella schultzei* head. Antennal segment III *Frankliniella* species **68–69**: **68** *fusca* **69** *cephalica* **70** *Frankliniella invasor* antenna. Tergite VIII *Frankliniella* species **71–76**: **71** *occidentalis* **72** *williamsi* **73** *crotalariae* **74** *hemerocallis* **75** *insularis* **76** *schultzei* **77** *Frankliniella williamsi* sternite II. *Kurtomathrips morrilli* **78–79**: **78** pronotum **79** tergites IV–V **80** *Limothrips cerealium* tergites VIII–X. *Microcephalothrips abdominalis* **81–82**: **81** prosternum **82** tergites IV & V.

**Figures 83–91.**
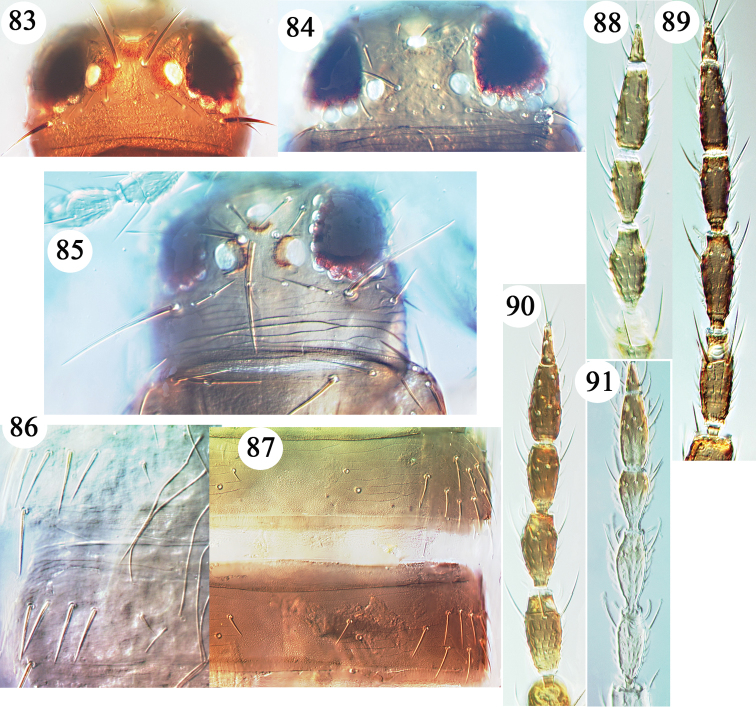
*Neurisothrips* species. Heads **83–85**: **83** *antennatus* holotype **84** *fasciatus* holotype **85** *fullawayi* holotype. Tergites IV–V **86–87**: **86** *carteri* holotype **87** *multispinus*. Antennae **88–91**: **88** *williamsi* holotype **89** *antennatus* holotype **90** *multispinus* **91** *dubauti*.

**Figures 92–110.**
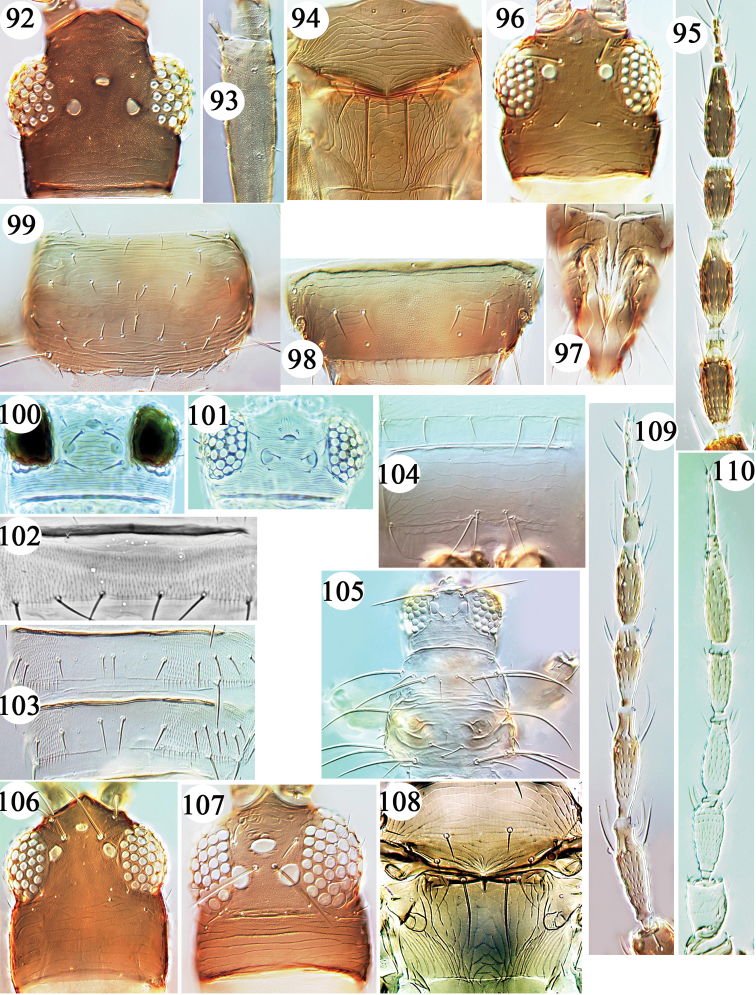
Thripinae from Hawaii. *Organothrips indicus* **92–93**: **92** head **93** fore tibia. *Pezothrips kellyanus* **94–95**: **94** meso & metanota **95** antenna. *Plesiothrips perplexus* **96–97**: **96** head **97** ovipositor. *Pseudanaphothrips araucariae* **98–99**: **98** tergite VIII **99** pronotum. *Scirtothrips* species **100–103**: **100** *citri* head **101** *dorsalis* head **102** *dorsalis* sternite IV **103** *inermis* tergites IV & V **104** *Rhamphothrips pandens* sternite VII **105** *Scolothrips sexmaculatus* head & pronotum. **106** *Sciothrips cardamomi* head **107** *Taeniothrips euchariae* head **108** *Tenothrips frici* meso & metanota.Antennae **109–110**: **109** *Psydrothrips kewi* **110** *Projectothrips bhattii*.

**Figures 111–132.**
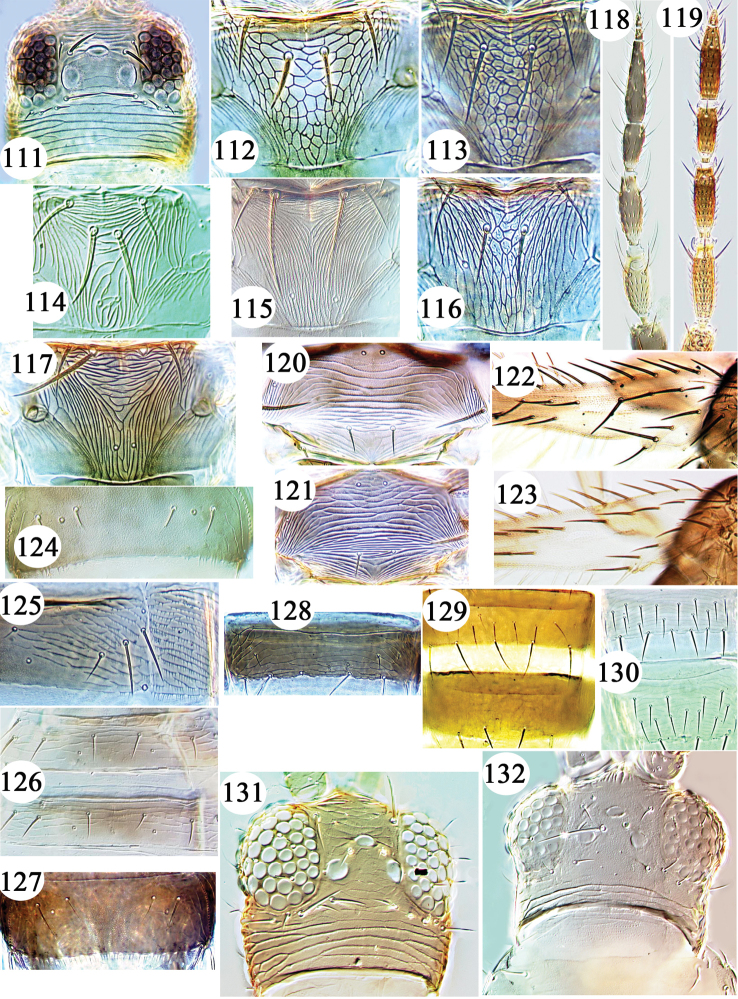
Thripinae from Hawaii. *Thrips* species **111–130**: **111** *hawaiiensis* head **112** *australis* metanotum **113** *orientalis* metanotum **114** *palmi* metanotum **115** *vittipennis* metanotum **116** *simplex* metanotum **117** *hawaiiensis* metanotum **118** *vittipennis* antenna **119** *australis* antenna **120** *florum* mesonotum **121** *hawaiiensis* mesonotum **122** *florum* clavus **123** *hawaiiensis* clavus **124** *vittipennis* tergite VIII **125** *tabaci* tergite & pleurotergite IV **126** *nigropilosus* tergites IV & V **127** *hawaiiensis* tergite VIII **128** *orientalis* sternite IV **129** *parvispinus* sternite VI & VII **130** *imaginis* sternites IV–V **131** *Stenchaetothrips minutus* head **132** *Trichromothrips cyperaceae* head.

### Key to suborders

**Table:** 

1	Abdominal segment X tubular; ovipositor inflatable and extruded at base of tube; fore wings when present, with no surface microtrichia nor visible longitudinal veins	**Tubulifera**
–	Abdominal segment X longitudinally divided ventrally, ovipositor of 4 saw-edged valves; fore wings, when present, with surface microtrichia and two longitudinal veins	**Terebrantia**

#### Sub-order Tubulifera

Worldwide in this suborder only one family (Phlaeothripidae) and two subfamilies (Idolothripinae and Phlaeothripinae) are recognised, although ThripsWiki (2015) provides a list of 14 more family-group names that have been proposed for particular groups of species. Both of the recognised subfamilies of Phlaeothripidae are represented in the Hawaiian fauna. Nishida et al. (1992) list 10 species of Idolothripinae and 46 species of Phlaeothripinae, whereas the Bishop Museum Hawaiian Arthropod Checklist (2015) lists 10 species of Idolothripinae and 50 species of Phlaeothripinae. Recent changes to the list of Phlaeothripinae (Mound and Okajima 2015) include one newly described species, *Dolichothrips franae* Mound & Okajima, and one name change with *Dolichothrips nesius* now a synonym of *Dolichothrips indicus*. Two genera dominate both lists of Phlaeothripinae: *Haplothrips* with nine species and *Hoplothrips* with 18 species. Some of the *Haplothrips* species cannot be recognised without a modern revision, and the situation with the species of *Hoplothrips* is even more unsatisfactory. Bagnall (1910) described 11 Hawaiian species that are now in *Hoplothrips*, and Mound (1968) studied the original specimens and concluded that it was not possible to determine how many separate species were represented. These specimens were re-examined in 2015, and there is little doubt that the number of real biological species is considerably less than eleven. However, formal synonymies cannot be proposed without field studies to establish the range of body size within at least one population, including the differences between large and small individuals, between sexes, and between winged and wingless morphs. The Phlaeothripidae of Hawaii are not considered further in this study.

#### Sub-order Terebrantia

Of the eight families worldwide that are recognised in this suborder (ThripsWiki 2015), only three have been found in the Hawaiian Islands. However, these include the two largest families that comprise the most common species of thrips around the world.

##### Key to Terebrantia families in Hawaiian Islands

**Table:** 

1	Antennal segments III and IV with emergent, simple or forked sense cones (Figs 1, 2)	**Thripidae**
–	Antennal segments III and IV with sensoria not protruding, elongate or oval (Figs 3, 6)	**2**
2	Antenna 9-segmented, III–IV elongate, parallel sided, with sensoria elongate along these segments (Figs 3, 4); fore wing broad, apex rounded (Figs 11–13)	**Aeolothripidae**
–	Antenna 8-segmented, III–IV with convex sides, sensoria transverse or sub-circular at apex of segments (Figs 6, 7); fore wing slender and pointed	**Merothripidae**

###### Aeolothripidae

Over 200 species in 23 genera are listed in this family (ThripsWiki 2015). More than 50% of these species are placed in the Holarctic genus *Aeolothrips*, and these are mostly flower-living but are possibly facultative predators. In contrast, a large proportion of Aeolothripidae genera are from the tropics, with many of the species obligate predators on other small arthropods in trees or at ground level.

####### Key to genera from Hawaiian Islands

**Table:** 

1	Antennal segment III less than 6 times as long as wide; sensoria on III–IV linear, no more than 0.5 as long as segment (Fig. 3)	***Aeolothrips***
–	Antennal segment III about 10 times longer than wide; sensoria on III–IV sinuous and almost as long as segment (Fig. 5)	***Franklinothrips***

######## AEOLOTHRIPS Haliday

The 105 species listed in this genus are almost entirely from the Holarctic, with no more than five species known from India or South Africa (ThripsWiki 2015). Bailey (1951) provided an introduction to the American species of the genus. One species of *Aeolothrips* is described from South America, but this cannot at present be distinguished from the widespread *fasciatus* that is also known from Hawaii.

######### Key to species from Hawaiian Islands

**Table:** 

1	Abdominal segments II and III pale (Fig. 11) in contrast to rest of blackish brown abdomen	***bicolor***
–	Abdomen completely blackish brown	**2**
2	Antennal segment VI less than one-half as long as V (Fig. 8)	***fasciatus***
–	Antennal segment VI about two-thirds as long as V (Fig. 9)	***nasturtii***

***Aeolothrips bicolor* Hinds**: This is considered to be a predator of scale insects and mites. Widespread in eastern USA southward through Texas and Mexico to Costa Rica, it has been found on Kauai, Maui, Molokai, and Oahu. The abdomen is bicoloured, the fore wing bears two dark cross bands, and tergite IX of the male bears a pair of claspers. It is ant-like in appearance and behavior, and is usually found living at ground level amongst grasses (Mound and Marullo 1996).

***Aeolothrips fasciatus* (Linnaeus)**: Described from Europe, this species appears to be a facultative predator of small arthropods in various flowers. It is recorded from Hawaii, Maui and Oahu, is widespread in USA, and is known from New Zealand and southern Australia. However, there are several species that are similar in colour and structure, and the precise identity of *fasciatus* cannot be established without males (Mound et al. 2012).

***Aeolothrips nasturtii* Jones**: This species is closely similar to *fasciatus* in appearance, apparently differing only in the relative proportions of the antennal segments, but unfortunately the male of *nasturtii* remains unknown. The species is recorded from Maui, and is widespread in USA into Canada.

######## Franklinothrips Back

This genus includes 16 species, and these occur in various countries around the tropics (Mound and Reynaud 2005). Most of the species seem to live solitary in the canopy of trees, where they are remarkable ant-mimics and predatory on other small arthropods.

***Franklinothrips vespiformis* Crawford**: Recorded widely around the world and often in vegetable crops, this species is reported from Hawaii and Oahu. It has sometimes been deployed as a biocontrol agent against pest thrips in European glasshouses (Loomans and Vierbergen 1999).

###### Merothripidae

This family comprises 16 species in three genera. A few species are found widely around the tropics, but most are Neotropical, with two species of one genus known from the Hawaiian Islands. Adult females bear a pair of lobes on the posterior margin of sternite VII, and these lobes have been considered to represent a reduced eighth sternite (Mound and O’Neill 1974).

####### Merothrips Hood

The species in this genus have 8-segmented antennae, with a circular or transverse sensorium at the apex of segments III and IV, and segment VIII elongate. They are minute insects that are fungus-feeders on dead twigs. The genus was treated by Zimmerman (1948) within the family Thripidae.

######## Key to species from Hawaiian Islands

**Table:** 

1	Pronotum with faint longtitudinal striae medially as well as near posterior margin	***morgani***
–	Pronotum with striae only on posterior area	***floridensis***

***Merothrips floridensis* Watson**: Widespread around the world, this species is recorded from Hawaii and Oahu. It is usually found on dead twigs and branches.

***Merothrips morgani* Hood**: Although collected much less frequently than *floridensis*, this species has also been reported widely around the world in warm areas on all continents. The synonym *hawaiiensis* Moulton was described from Hawaii, and *morgani* is recorded from Maui, Molokai and Oahu.

###### Thripidae

With rather more than 2000 species and 290 genera worldwide (ThripsWiki 2015), this is the second largest family of Thysanoptera. The species are almost entirely phytophagous on higher plants, both on leaves and in flowers, with a few predatory on mites, and a very few feeding on mosses or rarely on fungal pathogens of plants (Mound et al. 2012). Generally rather small in size, the members of this family are the organisms most commonly recognised as “thrips” by entomologists, and the family includes most of the species in this Order of insects that are considered pests.

####### Key to subfamilies of Thripidae

**Table:** 

1	Body surface strongly reticulate (Figs 16–18), particularly head, thorax and fore femora; usually dark brown in colour; fore wing with first longitudinal vein fused to costal vein; meso- and metathoracic furca with no spinula	**Panchaetothripinae**
–	Body surface either not reticulate, or with relatively weak sculpture, and colour varying from brown to almost white; fore wing first vein distinct from costa; meso- and/or metathoracic furca usually with median spinula	**2**
2	Metathoracic furca lyre-shaped, greatly prolonged anteriorly (Fig. 28)	**Dendrothripinae**
–	Metathoracic furca transverse or produced as simple median spinula (Figs 59–60)	**3**
3	Femora and tibiae with rows of microtrichia (Fig. 32); fore wing first vein with complete row of setae (Fig. 36), second vein with no setae or with only one or two near wing apex; sense cone on antennal segment VI with base greatly elongate and narrow; abdominal tergite IX without campaniform sensilla, with at least 4 pairs of posteromarginal setae	**Sericothripinae**
–	Femora and tibiae without closely spaced rows of microtrichia; fore wing chaetotaxy different; sense cone on antennal segment VI with base circular to elongate-oval; abdominal tergite IX usually with one or two pairs of campaniform sensilla (Fig. 80), with 2 or 3 pairs of posteromarginal setae	**Thripinae**

######## Panchaetothripinae

A taxonomic account of this group was provided by Wilson (1975), and it now comprises 135 species and 38 genera (ThripsWiki 2015). The species feed on green leaves where they usually pupate. Many are considered pests on various plants, but often as much from their habit of soiling leaves as of causing serious tissue damage. Most panchaetothripines live in tropical countries, but several are important pests in greenhouses in temperate regions.

######### Key to genera from Hawaiian Islands

**Table:** 

1	Fore wing with polygonal reticulation (Fig. 19); antenna 7-segmented	***Parthenothrips***
–	Forewing not reticulate; antenna 8-segmented	**2**
2	Antennal segments III–IV with simple sense cones (Fig. 23)	**3**
–	Antennal segments III–IV with sense cones forked (Fig. 1)	**6**
3	Abdominal segment II anterolaterally with dense cluster of recurved, stout, microtrichia (Fig. 24); mesonotum with complete longitudinal median division	**4**
–	Abdominal segment II without paired anterolateral clusters of stout microtrichia; mesonotum without complete longitudinal median division	**5**
4	Tergite X with apices of terminal setae acute; metanotum with longest pair of setae on anterior half of sclerite; tergites III–VII with paired clusters of reticles (Fig. 24)	***Anisopilothrips***
–	Tergite X with apices of terminal setal pair capitate; metanotum with longest pair of setae on posterior half of sclerite; tergites without clusters of reticles	***Elixothrips***
5	Fore wing anterior margin with cilia, posterior margin with cilia straight, wing apex rounded; head with polygonal reticulation, not projecting in front of eyes (Fig. 16)	***Heliothrips***
–	Fore wing anterior margin without cilia, posterior margin with cilia wavy, wing apex pointed; head projecting in front of eyes, with rugose sculpture (Fig. 25)	***Rhipiphorothrips***
6	Fore wing uniformly deeply shaded (Fig. 20); head with a basal neck; abdominal tergite X without dorsal split	***Selenothrips***
–	Fore wing not uniformly dark; head without distinct neck; abdominal tergite X with dorsal longitudinal split	**7**
7	Tarsi 2-segmented; fore wing with 2 complete rows of setae (Fig. 21)	***Hercinothrips***
–	Tarsi one-segmented; fore wing first vein with few setae (Fig. 22).	**8**
8	Head without markings within sculptured reticles, except posterior to strong occipital ridge (Fig. 17); abdominal tergites with scalloped antecostal ridge (Fig. 26)	***Helionothrips***
–	Head with markings within sculptured reticles, without occipital ridge (Fig. 18); abdominal tergites with antecostal ridge not scalloped (Fig. 27)	***Caliothrips***

########## ANISOPILOTHRIPS Stannard & Mitri

Only one species is recognized in this tropical genus. It is closely related to *Elixothrips* and also to *Astrothrips* in having a dense cluster of recurved microtrichia on the second abdominal tergite.

***Anisopilothrips venustulus* (Priesner)**: Described from, and widespread around, the Caribbean, this species is also recorded from Florida, as well as Taiwan, Japan, Australia and Fiji. Abdominal tergites III–VII each have a conspicuous pair of reticulate areas arranged like a flower or bunch of grapes (Fig. 24). The species is listed from Hawaii without further data in an unpublished, updated list of Hawaiian insects provided by Bernarr Kumashiro, and has been intercepted from Hawaii by quarantine officials in California.

########## CALIOTHRIPS Daniel

This genus of 22 species is widespread around the tropics, and Nakahara (1991) provided a key to 10 species from the Nearctic region. Leaf-feeding at all stages, but pupating at ground level, a few species are considered crop pests, including of cotton seedlings in parts of Africa (Wilson 1975). The hind coxae of adults have a coiled apodeme internally, and membership of the genus is usually obvious because of the presence of many markings within the sculptured reticles on the head and pronotum. Both species recorded from Hawaii have the lateral areas of the tergites with polygonal reticulation.

########### Key to species from Hawaiian Islands

**Table:** 

1	Fore wing brown with 2 white crossbands (Fig. 22); antennal segments III–IV yellow with brown median area, V brown with base yellow	***fasciatus***
–	Fore wing white except for brown apex and occasional pale brown area in basal quarter; antennal segments III–V predominantly yellow, with apex pale brown	***punctipennis***

***Caliothrips fasciatus* (Pergande)**: The North American Bean Thrips, this species used to be considered a pest of agricultural crops in western USA (Bailey 1957), but is now considered of limited importance (Hoddle et al. 2006). It has been reported from Hawaii, Lanai, Molokai, and Oahu, as well as Canada, Mexico, Puerto Rico, Argentina, and Brazil, but an old record from China is possibly an error (Mound et al. 2011).

***Caliothrips punctipennis* (Hood)**: Little is known about this American grass-living species. It has curiously pale fore wings, and is recorded from Florida, Texas and Mexico, as well as Maui and Oahu.

########## ELIXOTHRIPS Stannard & Mitri

The only species in this genus bears an array of recurved stout microtrichia anterolaterally on the second abdominal tergite. In this it resembles species of the tropical genera *Astrothrips* and *Anisopilothrips*, but is distinguished by the presence of a pair of capitate setae at the abdominal apex.

***Elixothrips brevisetis* (Bagnall)**: Described from the Seychelles, this species has been reported widely from countries bordering the Pacific Ocean, as well as from Hawaii and Oahu. It is a pest of banana fruits in Martinique as well as Hawaii (Muruvanda 1986), but the male of the species remains unknown.

########## HELIONOTHRIPS Bagnall

A total of 27 species from various tropical countries are currently listed under this genus (ThripsWiki 2015), and a few of these are sometimes reported as minor pests (Wilson 1975). They are characterized by the sculpture of the antecostal ridge on the abdominal tergites that forms a series of prominent scallop-like ridges (Fig. 26).

***Helionothrips errans* (Williams)**: Described from a greenhouse in Europe, this species is widespread in tropical countries, and is commonly considered a pest of cultivated orchids, including *Cymbidium* species. It has been reported from the Hawaiian Islands only once, from a greenhouse in the Volcano area of Hawaii.

########## HELIOTHRIPS Haliday

Three species are now considered valid in this genus, all from South America (Nakahara et al. 2015). Two of the species have restricted distributions in southern Brazil, but the third is found worldwide.

***Heliothrips haemorrhoidalis* (Bouché)**: The Greenhouse thrips is a common minor pest, but can be particularly damaging on plants that are growing suboptimally or where fruit hanging in clusters touch each other and provide protected areas for larvae, pupae, and adults to conceal themselves. Mature adults are dark brown with yellow legs, but adults freshly emerged from pupae commonly have the abdomen golden yellow. Attacked plants usually bear distinctive “bleached” areas that result from feeding and small black faecal spots that larvae and adults deposit on leaves. The host-plant range is extensive, but commonly involves plants with hard leaves that lack glandular trichomes (Scott-Brown and Simmonds 2006). Originally from Southwestern Brazil but now cosmopolitan, this thrips is recorded from Hawaii, Kauai, Maui, Midway, Molokai, and Oahu.

########## HERCINOTHRIPS Bagnall

The nine species included in this African genus are unique amongst Panchaetothripinae in having the tarsi two segmented, and the fore wings with both longitudinal veins bearing a complete row of setae. Two of the species are widespread around the world, but a third has recently been recorded as introduced to Portugal, damaging the leaves of succulent plants of the genus *Aloe* (Mateus et al. 2015).

########### Key to species from Hawaiian Islands

**Table:** 

1	Fore wing median pale band longer than the brown cross bands on either side (Fig. 21)	***bicinctus***
–	Fore wing median pale band indistinct or shorter than dark areas on either side	***femoralis***

***Hercinothrips bicinctus* Bagnall**: Widespread throughout subtropical countries, and sometimes common in greenhouses in temperate countries, this species was first collected on the Hawaiian Islands at Pauoa, Oahu, 16.vi.1998, from *Coccinea grandis*. Highly polyphagous, it is often a pest on banana fruits.

***Hercinothrips femoralis* (Reuter)**: Similarly polyphagous and as widespread around the world as *bicinctus*, this species is reported from Hawaii, Kauai, Maui, and Oahu.

########## PARTHENOTHRIPS Uzel

Only one species is placed in this genus, and this is easily recognised from the highly distinctive broad reticulate fore wings (Fig. 19). The country of origin of the genus remains in doubt, although amongst Panchaetothripinae only *Arachisothrips* from the Neotropics has such a broad fore wing.

***Parthenothrips dracaenae* (Heeger)**: Commonly known as the Parlour Palm thrips, from its worldwide association with the leaves of cultivated *Howea* palms, this species is not commonly taken out of doors. On the Hawaiian Islands it has been taken on the leaves of *Cordyline terminalis* and *Polyscias* sp., and is recorded from Hawaii, Kauai, Maui, and Oahu.

########## RHIPIPHOROTHRIPS Morgan

This genus comprises three Asian and two African species (Wilson 1975). The head bears rugose, irregular reticulate sculpture, with a distinctive transverse ridge, and a prolongation in front of the eyes (Fig. 25). The fore wings have minute veinal setae, and lack cilia on the costal margin.

***Rhipiphorothrips pulchellus* Morgan**: Distinctively coloured, with the head and sides of the pterothorax brown, but the pronotum, abdomen, antennae and legs yellow, this species was decribed originally from Sri Lanka. It is widely recorded across Asia, from a range of different plant species. In contrast to a very similar Asian species, *cruentatus*, the males do not have a tooth-like tubercle laterally on the fourth abdominal tergite. It has been found on Oahu, in association with *Acalypha wilkesiana*, *Carissa grandiflora*, *Eugenia malaccensis*, *Leucaena leucocephala*, and *Mangifera indica*.

########## SELENOTHRIPS Karny

Only one species is placed in this genus. It is readily recognised from the reticulate head sharply constricted to a basal neck that lacks reticulation, and the unusually dark fore wings that have two rows of widely spaced black setae (Fig. 20).

***Selenothrips rubrocinctus* (Giard)**: Known as the red-banded cacao thrips, because of its brightly coloured larvae and frequent association with cocoa tree foliage, this species is polyphagous. Probably Neotropical in origin, this species is now pantropical, and has been recorded from Hawaii, Kauai, Lanai, Maui, Molokai, and Oahu, on the leaves of a wide range of unrelated plant species.

######## DENDROTHRIPINAE

Worldwide, there are almost 95 species listed in 12 genera in this subfamily (ThripsWiki 2015). Mostly from the Old World with few listed from the Americas, they seem to be mainly associated with the leaves of trees. Only two genera are recorded from the Hawaiian Islands. All species in this subfamily have a greatly enlarged lyre-shaped metafurca (Fig. 28) that extends to the mesothorax, and is associated with large muscles that facilitate the remarkable jumping ability of these thrips.

######### Key to genera from Hawaiian Islands

**Table:** 

1	Abdominal tergites III–VI with median pair of setae small and wide apart (Fig. 30); pronotum with no long setae; ocellar setae pair III between posterior ocelli	***Asprothrips***
–	Abdominal tergites III–VI with median pair of setae longer than distance between their bases (Fig. 31); pronotum with 2 pairs of long posteroangular setae; ocellar setae pair III arising anterior to ocellar triangle	***Leucothrips***

########## ASPROTHRIPS Crawford

Five species are recognised in this genus, and these are probably all East Asian in origin. In contrast to the single species recorded from Hawaii, the others all have the body dark brown at least in part.

***Asprothrips seminigricornis* (Girault)**: This is a minute, white thrips with the terminal antennal segments dark brown. It has been described under three different names, from Australia, New York and Hawaii, the latter name being *Scirtothrips antennatus*. It is highly polyphagous, and in Australia has been found breeding on the young leaves of peach (*Prunus persica*), Tung Tree (*Vernicia fordii*), granadilla (*Passiflora ligularis*), mulberry (*Morus alba*), and also *Citrus* (Mound and Wells 2015). It is also recorded from California, Florida, and Bermuda, as well as Hawaii, Kauai, and Oahu.

########## LEUCOTHRIPS Reuter

Four of the five known species in this genus are from the New World, one from Guadeloupe, two from South America and one from North and South America. The fifth species, *nigripennis*, is widespread around the world in association with cultivated ferns, and is readily recognized by its uniformly dark fore wings. The genus seems to be the ecological equivalent of the Eurasian genus *Dendrothrips*, living on the leaves of a range of trees and herbs. The species of these two genera share a curious instability in antennal segmentation and form of sense cones. *Leucothrips* species have the fore wing costal cilia arising at the margin, although *Dendrothrips* species have these cilia arise submarginally and ventrally.

########### Key to species from Hawaiian Islands

**Table:** 

1	Antennal segments III and IV with sense cone simple in both sexes; head pale	***piercei***
–	Antennal segments III and IV with sense cone forked in female, simple in male; head with small red spot on anterior margin at base of antennae	***theobromae***

***Leucothrips piercei* (Morgan)**: Described originally from Texas, this species is recorded from Arizona and California, as well as Mexico, Surinam, Brazil and Peru. It is known from Kauai and Oahu, where it is common on leaves of *Xanthium*, and also on beets, bean, carrot, eggplant, lettuce, mustard, parsley, *Hibiscus*, and radish (Zimmerman 1948).

***Leucothrips theobromae* (Priesner)**: Described from Surinam, this species seems to be widespread in at least the northern parts of South America, including Panama, Colombia, Ecuador and Peru. On Oahu it has been taken from the leaves of *Ricinus communis*.

######## SERICOTHRIPINAE

Currently three genera are recognised in this subfamily of 152 species. However, a further 12 generic names are available that have been proposed for individual species with one or more exceptional characteristics (ThripsWiki 2015). The phylogenetic significance of the three genera remains doubtful. One of them, *Hydatothrips*, occurs only in warmer parts of the world, whereas the other two are more widespread.

######### Key to genera from Hawaiian Islands

**Table:** 

1	Metanotal sculpture longitudinal, with no microtrichia (Fig. 34)	***Neohydatothrips***
–	Metanotal sculpture transverse, posterior third with rows of microtrichia (Fig. 35)	***Sericothrips***

########## NEOHYDATOTHRIPS John

Worldwide, a total of 103 species is recognised in this genus, and these species occur in both temperate and tropical zones of both hemispheres. Members of this genus are fully winged, and the metasternal anterior margin is transverse with only a small indentation. Many of the species are brightly coloured with various patterns of dark and light on the body and fore wings. There are many microtrichia on the lateral thirds of the tergites, and the fore wing has a complete row of setae on the first vein (Fig. 36) but no setae on the second vein except sometimes near the wing apex. Only two species are known from the Hawaiian Islands, and both are widespread around the world.

########### Key to species from Hawaiian Islands

**Table:** 

1	Fore wing almost uniformly pale or lightly shaded, clavus darker (Fig. 37); body mainly yellow; on *Sida* spp.	***gracilipes***
–	Fore wing with two dark bands; body mainly dark brown (Fig. 33); on *Tagetes* spp.	***samayunkur***

***Neohydatothrips gracilipes* (Hood)**: Although adults are recorded from various plants, this species seems to breed particularly on small weeds in the genus *Sida*, but possibly also on other Malvaceae. The legs and body are mainly yellow, but there are small brown markings on the head and thorax, and the tergal antecostal ridges are dark. The male bears a single small pore plate on the seventh sternite. Described from Mexico, this thrips is recorded from Maui and Oahu, and is known from many other areas including Texas, Trinidad, Costa Rica, India and Australia.

***Neohydatothrips samayunkur* (Kudo)**: Apparently specific to species of *Tagetes*, this thrips has probably been dispersed by the horticultural trade in marigold plants. Recorded from Hawaii and Oahu, it has been seen from Florida, Mexico, Costa Rica, El Salvador, Brazil, Japan, Australia, Kenya, and Sri Lanka.

########## SERICOTHRIPS Haliday

Eight species are included in this genus, one of which is from South Africa and the others from the Northern Hemisphere. Unlike the members of the other two genera in this subfamily, the species of *Sericothrips* have a tendency to produce apterous adults.

***Sericothrips staphylinus* Haliday**: This European thrips was introduced to Hawaii as a biological control agent against the knoxious shrubby weed *Ulex europaeus* (Markin et al. 1996), on which it has become established. Adults of both sexes are usually wingless, with macropterous females relatively uncommon.

######## THRIPINAE

This is the largest of the Terebrantia subfamilies, with nearly 1700 species in 235 genera. The group is represented throughout the world, wherever green plants can grow, and many species are considered to be crop pests. A particularly large number of taxa are specific to Poaceae (Mound 2011b), with smaller numbers associated with Fabaceae and Orchidaceae, but host-plant exploitation by these insects seems to have been largely opportunistic (Mound 2004), with many species in the largest genera (*Thrips* and *Frankliniella*) being polyphagous.

######### Key to genera from Hawaiian Islands

**Table:** 

1	Antennal segments III–IV each with a simple sense cone (Figs 2, 53)	**2**
–	Antennal segments III–IV each with a forked sense cone (Figs 1, 70)	**11**
2	Female tergite X with prominent pair of thorn-like setae (Fig. 80)	***Limothrips***
–	Female tergite X without such stout setae	**3**
3	Antennal segment II strongly asymmetric, external margin produced into a point (Figs 2, 37); pronotum trapezoidal, posterior margin wider than anterior (Fig. 37)	**4**
–	Antennal segment II more or less symmetrical, not produced laterally; pronotum transverse (if rarely trapezoidal then with prominent flattened pair of setae at posterior angles: Fig. 78)	**5**
4	Mesothoracic sternal furca strongly developed with paired lateral flanges and invaginated medially (Fig. 54); antennal segment I less than 2.5 times as wide as base of segment II	***Chirothrips***
–	Mesothoracic sternal furca weakly developd, without paired lateral flanges, invaginations widely separated (Fig. 55); antennal segment I more than 2.5 times as wide as base of segment II (Fig. 37)	***Arorathrips***
5	Head clearly longer than wide but not projecting in front of eyes (Fig. 41); body yellow; antennae 6-segmented (Fig. 40)	***Aptinothrips***
–	Head different; body usually brown; antennae 8- or 9-segmented	**6**
6	Abdominal tergites VI–VIII with paired ctenidia laterally (Fig. 46)	***Bolacothrips***
–	Abdominal tergites without paired ctenidia	**7**
7	Fore wing costa and first vein each with row of long capitate setae (Fig. 63), pronotum with two pairs of similar capitate posteroangular setae; fore wing second vein without setae	***Echinothrips***
–	Fore wing without such long capitate setae, or wing absent	**8**
8	Head prolonged in front of eyes (Fig. 92); fore tibia with fringed spur at inner apex (Fig. 93)	***Organothrips***
–	Head and fore tibia different	**9**
9	Pronotum with no long setae; sternites with deeply lobed craspedum (Fig. 38); dark brown apterous species	***Apterothrips***
–	Pronotal posteroangular setae prominent; sternites without lobed craspeda	**10**
10	Pronotum with two pairs of long pointed posteroangular setae; tergal median setae small and far apart, craspedum with rounded lobes (Fig. 47)	***Bregmatothrips***
–	Pronotum with one pair of long, flattened posteroangular setae (Fig. 78); tergal median setae long and close together, craspedum with pointed teeth (Fig. 79)	***Kurtomathrips***
11	Lateral thirds of tergites with many irregularly arranged, broadly based, stout microtrichia (Fig. 56); head with prominent postocular ridge	***Dendrothripoides***
–	Tergites and head different	**12**
12	Lateral thirds of tergites with closely spaced rows of small microtrichia (Fig. 103)	***Scirtothrips***
–	Lateral thirds of tergites without or with few irregular microtrichia	**13**
13	Female sternite VII with posteromarginal setae pairs I and II close together medially and distant from lateral pair S III (Fig. 104)	***Rhamphothrips***
–	Female sternite VII with all three pairs of posteromarginal setae equidistant from each other	**14**
14	Female with ovipositor weak and lacking serrations or with weak serrations (Fig. 97); male antenna with segment III very small but IV–VI greatly enlarged	***Plesiothrips***
–	Female with ovipositor bearing conspicuous serrations, extending to apex of segment X	**15**
15	Pronotum without prominent posteroangular setae (Fig. 57)	**16**
–	Pronotum with one or two pairs of prominent posteroangular setae (Figs 65, 99)	**18**
16	Tergites II–IV with median setal pair long and close together (Fig. 45); tergal posterior margin with craspedum; sternites with discal setae and 4–5 pairs of posteromarginal setae; male sternite III with glandular opening on antecostal region	***Baileyothrips***
–	Tergal median setae arising further apart from each other than their length; tergites without any craspeda; sternites without discal setae, with 3 pairs of marginal setae; male with sternal pore plates	**17**
17	Ocellar setae pair I present; antennal segment VI with partial division giving apparent 9-segmented condition (Fig. 39), III and IV not prolonged into an apical neck	***Anaphothrips***
–	Ocellar setae pair I absent; antennae 8-segmented, III and IV with elongate apex and sense cones extending more than one third across succeeding segment (Fig. 58)	***Dichromothrips*** [in part]
18	Tergite VIII with paired ctenidia anterolateral to spiracle (Figs 71, 76)	**19**
–	Tergite VIII *either* without ctenidia *or* with paired ctenidia posteromesad of spiracle (Fig. 15)	**20**
19	Pronotum posterior margin with one pair of small setae between the major median posteromarginal setae (Fig. 65); tergites V–VI with well-developed pairs of ctenidia laterally; ocellar setae pair III arising between or in front of posterior ocelli	***Frankliniella***
–	Pronotum posterior margin without pair of small setae between major median pair of posteromarginal setae (Fig. 99); tergites V–VI without well-developed ctenidia laterally; ocellar setae pair III arising on tangent between posterior margins of posterior ocelli	***Pseudanaphothrips***
20	Tergites V–VII with paired ctenidia (Fig. 15); head without pair of setae in front of first ocellus (Fig. 111)	**21**
–	Tergites V–VII without any ctenidia; head usually with pair of setae in front of first ocellus (Fig. 85)	**23**
21	Prosternum with several pairs of setae (Fig. 81); tergites with toothed craspedum (Fig. 82)	***Microcephalothrips***
–	Prosternum without setae; tergites without prominent craspedal teeth	**22**
22	Ocellar setae pair II longer than pair III (Fig. 131)	***Stenchaetothrips***
–	Ocellar setae pair II shorter than or equal to pair III (Fig. 111)	***Thrips***
23	Pronotum with 6 pairs of very long setae (Fig. 105)	***Scolothrips***
–	Pronotum with no more than three pairs of long setae	**24**
24	Antennal segment VIII spindle-shaped, almost 4 times as long as VII (Fig. 110); pronotum with 3 pairs of long posteroangular setae	***Projectothrips***
–	Antennal segment VIII shorter (Figs 88–91); no more than 2 pairs of pronotal posteroangular setae	**25**
25	Fore wing first and second longitudinal veins both with complete row of setae	***Neurisothrips***
–	Fore wing veins with incomplete setal rows	**26**
26	Tergite VIII with area of specialised sculpture extending anteromesad from spiracles (Fig. 48)	***Chaetanaphothrips***
–	Tergite VIII without such specialised sculpture around spiracles	**27**
27	Head with ocellar setae pair I present	**28**
–	Head lacking ocellar setae pair I in front of first ocellus (Figs 106–107)	**30**
28	Antenna with 9 segments (Fig. 109)	***Psydrothrips***
–	Antenna with 8 segments (Fig. 98)	**29**
29	Mesonotum with median setal pair arising in front of posterior margin (Fig. 108); ocellar setae pair III arise anterolateral to ocellar triangle; antennal III largely yellow	***Tenothrips***
–	Mesonotal median setal pair arising at posterior margin (Fig. 94); ocellar setae pair III arise within ocellar triangle; antennal segment III brown with constricted apical third white (Fig. 95)	***Pezothrips***
30	Tergite VIII with no posteromarginal comb	**31**
–	Tergite VIII with complete posteromarginal comb of microtrichia (Fig. 61)	**32**
31	Fore wing second vein with few, widely spaced setae; tergite IX of female with campaniform sensilla; male without sternal pore plates, and tergite IX without drepanae	***Danothrips***
–	Fore wing second vein with complete setal row; tergite IX of female without campaniform sensilla; male with sternal pore plates and tergite IX with pair of drepanae	***Trichromothrips***
32	Metathoracic furcal spinula present but weak in *corbetti* (Figs 59–60); on Orchidaceae	***Dichromothrips***
–	Metathoracic furca with no spinula	**33**
33	Head prolonged in front of compound eyes (Fig. 106); fore wing second vein with 4 or 5 widely spaced setae	***Sciothrips***
–	Head not prolonged in front of eyes (Fig. 107); fore wing second vein setal row continuous	***Taeniothrips***

########## ANAPHOTHRIPS Uzel

More than 80 species are listed in this genus. In the northern hemisphere many of the species are associated with Poaceae (Nakahara 1995), but in Australia members of this genus are known to breed on plants in a wide range of other families (Mound and Masumoto 2009). Adults have no long setae on the pronotum, and males in many species have C-shaped sternal pore plates. Both of the species recorded from the Hawaiian Islands have a partial suture across antennal segment VI (Fig. 39), thus producing a 9-segmented condition that is unusual amongst Thripinae.

########### Key to species from Hawaiian Islands

**Table:** 

1	Abdominal tergites with many sculpture lines across median area; tergite IX major setal pairs S1 and S2 not extending beyond apex of segment X.	***obscurus***
–	Abdominal tergites with no lines of sculpture on median area; tergite IX major setal pairs S1 and S2 extending beyond apex of segment X	***swezeyi***

***Anaphothrips obscurus* (Müller)**: Widely distributed throughout the temperate areas of the world on various Poaceae, this yellow thrips is sometimes a minor pest of cereal crops, and has been taken on Maui and Oahu. The male is known only from Iran (Mirab-Balou and Chen 2010), the worldwide populations comprising only females.

***Anaphothrips swezeyi* Moulton**: Although this small yellow species was described originally from sugar cane in the Hawaiian Islands, there is no evidence that this is where it originated. It has been recorded from Hawaii, Kauai, Lanai, Molokai and Oahu, but was taken commonly on New Caledonia, and also along the coastal region of eastern Australia from where the male was first described (Mound and Masumoto 2009).

########## APTEROTHRIPS Bagnall

Only two species are placed in this genus, and Nakahara (1988) provided details of how to distinguish them. They are both dark brown, wingless species, with the antennae 9-segmented due to an incomplete suture across segment VI, and the sternites bear a lobed craspedum on the posterior margin (Fig. 38).

***Apterothrips apteris* (Daniel)**: Described from California, this species is found southwards along the west of South America, being recorded from Mexico, Panama, Ecuador, Chile, Peru and southern Argentina, and then across the Southern Ocean to New Zealand and Australia. In Hawaii it was originally misidentified and recorded as *secticornis*, a species that occurs northwards from California and in northern Europe. In coastal California, *apteris* breeds on *Erigeron*, but in Western Australia and New Zealand it has caused damage to alfalfa, and in Tasmania it has been found on garlic plants (Mound and Wells 2015).

########## APTINOTHRIPS Haliday

Four species are recognised in this genus, all wingless (Fig. 41) and living on grass leaves, and all were originally native to northern Europe. Two species are now widely distributed around the world, and *rufus* is probably one of the most common insects worldwide.

***Aptinothrips rufus* (Haliday)**: The form of the 6-segmented antennae, with the large terminal segment, is distinctive in this species (Fig. 40). Living on the leaves of various Poaceae, it is recorded from Hawaii, Maui and Oahu, and occurs in temperate climate zones around the world, including highland areas in tropical countries such as Kenya and Costa Rica.

########## ARORATHRIPS Bhatti

This genus comprises 16 species and was erected for a series of species originally placed in the genus *Chirothrips*. As with the members of that genus, all of the species breed in the flowers of Poaceae. Nakahara and Foottit (2012) provided an account of *Arorathrips* species, all of which are from the Americas, although *mexicanus* is widespread around the tropics.

########### Key to species from Hawaiian Islands

**Table:** 

1	Outer margin of fore tibia prolonged around base of second tarsal segment (Fig. 37); abdominal tergites and sternites with tuberculate scallops on anterior margin (Fig. 43)	***mexicanus***
–	Outer margin of fore tibia not prolonged; tergites and sternites without tuberculate scallops.	**2**
2	Head with at least 30 small setae; prosternal basantra with setae; abdominal sternites with median 2 pairs of posteromarginal setae arising on posterior margin	***fulvus***
–	Head with 10–22 small setae (Fig. 42); basantra without setae; abdominal sternites with median 2 pairs of setae arising in front of posterior margin	***spiniceps***

***Arorathrips fulvus* (Moulton)**: Described originally from Oahu, with one synonym from Brazil, this species is recorded from Texas, Tennessee, Argentina, and Uruguay, as well as Hawaii and Kauai. Yellowish-brown, it sometimes has the abdomen paler than the thorax, and is unusual in having many small setae on the prosternal basantra. It is possibly associated with species of *Paspalum*.

***Arorathrips mexicanus* (Crawford)**: In addition to the original description based on specimens from Mexico, this species has been described under three other names, from Florida, Louisiana and Argentina. It is widespread in the Americas, and known from South Africa, Thailand, Philippines, Australia and New Caledonia, as well as Hawaii, Kauai, Lanai, Maui, Midway and Oahu. It is one of the most common thrips on grasses in many tropical areas, and is reported from various species of tropical Poaceae, including sugar cane.

***Arorathrips spiniceps* (Hood)**: Described from Arizona, with one synonym from Hawaii, this species is widespread across the USA, and is recorded from Mexico, Guatemala, Cuba, Costa Rica, Dominica, Bermuda, Argentina, Papua, Solomon Islands and Australia, as well as Kauai and Oahu. It is recorded from various species of Poaceae, including sugar cane.

########## BAILEYOTHRIPS Kono & O’Neill

Only two species are recognized in this genus, although doubt has been expressed that these are distinct species (Mound and Marullo 1996). These thrips originated, presumably, from southern USA or the Carribean region. Originally described as species of *Anaphothrips*, due to the lack of long pronotal posteroangular setae, they differ from that genus in having tergites with a posteromarginal craspedum that is toothed laterally, and the sternites with discal setae.

########### Key to species from Hawaiian Islands

**Table:** 

1	Abdominal tergites laterally with many discal microtrichia (Fig. 45); pronotum without brown spots; antennal segments IV–V grayish brown with bases pale, VI brown	***arizonensis***
–	Abdominal tergites with few discal microtrichia except on anterior tergites; pronotum with light brown spots in submarginal row on each side, antennal segments IV–V pale in basal half, base of VI occasionally pale	***limbatus***

***Baileyothrips arizonensis* (Morgan)**: Described from Arizona with one synonym from California, this species is recorded from Texas and Florida as well as Oahu. Reported from various plants, it appears to be associated species of *Euphorbia*, although Bailey (1940) indicated that it was a minor pest of cotton.

***Baileyothrips limbatus* (Hood)**: As indicated above, this species is very similar to *arizonensis*, and is also associated with *Euphorbia* species. Described from Panama, it is recorded from Costa Rica, Guatemala, Jamaica, Trinidad and Florida, as well as Maui.

########## BOLACOTHRIPS Uzel

Twelve species are listed in this genus, and with one exception, all of them are from tropical and subtropical countries living on Poaceae. It is closely related to the genus *Thrips*, in lacking ocellar setae pair I and in having tergal ctenidia with those on VIII posteromesad of the spiracles (Fig. 46). However, the sense cones on antennal segments III and IV are simple, not forked (Mound 2011b).

***Bolacothrips striatopennatus* (Schmutz)**: Described from Sri Lanka, and known on grasses from India to Japan and Taiwan, also Guam, northern Australia, Florida, Georgia and Kauai.

########## BREGMATOTHRIPS Hood

The nine species currently recognized in this genus are from various warm temperate to subtropical parts of the world, all living on Poaceae, with *venustus* widely distributed. The females have a narrow pale, weakly scalloped, craspedum on the posterior margin of the tergites (Fig. 47) and they all have two dorso-apical setae on the first antennal segment, except *venustus* that has a single such seta An identification key to the species was provided by Mound (2011b).

***Bregmatothrips venustus* Hood**: This species was recorded from Oahu under the synonymic name *sonorensis* Stannard (see Bhatti 1984). It is recorded from various grasses, including *Echinochloa crus-galli*, and is widespread across southern USA to Mexico and Panama. Winged females are brown in colour, but the short-winged females and males are bicoloured with most of the thorax yellow.

########## CHAETANAPHOTHRIPS Priesner

This is a Southeast Asian genus of 20 species, of which three species are widespread around the world, mainly in the tropics but sometimes in greenhouses in temperate areas. All the species are yellow, with a characteristic area of stippled cuticle extending anteromesad from the two spiracles on tergite VIII (Fig. 48), and the males, where known, have a pair of large, stout setae on tergite IX that arise from a common tubercle.

########### Key to species from Hawaiian Islands

**Table:** 

1	Head with 3 pairs of ocellar setae, ocellar setae pair I present anterolateral to first ocellus (Fig. 49); female with sternite II lacking discal setae, and sternite III with a small pore plate (Fig. 50)	***signipennis***
–	Head with 2 pairs of ocellar setae, ocellar setae pair I absent; sternite II of female with 1–3 discal setae, sternite III without a pore plate	**2**
2	Pronotum with 2 pairs of posteroangular setae, outer pair shorter than inner pair; fore wing with median brown band long, occupying at least half of wing length (Fig. 52)	***orchidii***
–	Pronotum with 1 pair of short posteroangular setae; fore wing with median brown band short, 1–3 times as long as width of wing	***leeuweni***

***Chaetanaphothrips leeuweni* (Karny)**: This appears to be the least common of the three widespread pest species. It is recorded widely in the Caribbean islands, also Florida, and on several Pacific islands including Oahu.

***Chaetanaphothrips orchidii* (Moulton)**: This species is a polyphagous leaf feeder in moist environments and is an occasional pest of *Anthurium*, orchids, and *Asystasia* in Hawaii. It induces and feeds within rolled leaves (Mound and Wells 2015), young terminals, flower buds and under basal leaf sheaths of petioles, causing scarring and malformation of leaves and flowers, and feeding may also retard plant growth (Sakimura 1975). Sometimes reported as a pest of banana fruits, orchid flowers, and ornamental plants in greenhouses, this is one of three thrips species causing ringspots on oranges and grapefruits in Florida (Childers and Frantz 1994). Reported from Hawaii, Kauai, Maui, and Oahu, it is widespread around the world.

***Chaetanaphothrips signipennis* (Bagnall)**: Known as the banana rust thrips, this species is known as a pest of bananas in Oriental, Australian, and Central American regions, and causes white streaks in Hawaii on Ti leaves, *Cordyline terminalis* (Sakimura 1975). Recorded from Hawaii and Oahu, it is known from many tropical and subtropical countries, including Florida. It is unusual within this genus in the presence of ocellar setae pair I, and also the presence of a pore plate on the third sternite in females.

########## CHIROTHRIPS Haliday

There are 42 species currently listed in this genus, mostly from temperate parts of the world. As with the species of *Arorathrips*, these thrips breed within the florets of Poaceae, usually with only one larva in each floret (Nakahara and Foottit 2012). Moreover, they pupate within the glumes, and many species are thus distributed in commercial grass seed. They have a characteristic body form, with the pronotum trapezoidal, the head rather small, the fore legs rather stout, and the antennae short with the second segment asymmetric.

########### Key to species from Hawaiian Islands

[* doubtful record]

**Table:** 

1	Antennal segment II strongly produced laterally with margin concave (Fig. 2); sternites with craspedum bearing rounded teeth	***manicatus*** *
–	Antennal segment II asymmetric, angulate laterally with margin almost straight; sternites with craspedum bearing conical teeth	***patruelis***

***Chirothrips manicatus* (Haliday)**: No specimens of this species have been seen from the Hawaiian Islands, despite the record in Nishida et al. (1992). It is included here primarily because it is one of the most common Thysanoptera species in the northern temperate zone, and is likely to occur in Hawaii. A European insect, it is also known from southern Australia and New Zealand. The species breeds in the flowers of grasses, the larvae having reduced legs and unable to move from one floret to another. It is recorded as a pest of cereal crops (Lewis 1973) but breeds in many Poaceae.

***Chirothrips patruelis* Hood**: Described originally from New York, this species is widespread from Canada to New Mexico and Arizona, but is also recorded from Peru and Hawaii. It is similar in structure to *manicatus*, and presumably has similar relationships to grass flowers.

########## DANOTHRIPS Bhatti

Nine species are listed in this Southeast Asian genus. It is presumably related to *Chaetanaphothrips*, the male having a pair of spine-like setae medially on tergite IX, but the tergites and sternites lack craspeda, and there is no comb on tergite VIII.

***Danothrips trifasciatus* Sakmura**: Adults of this small yellow species with banded fore wings have been collected from a wide range of unrelated plants. It has been reported as causing leaf damage to *Anthurium*, also ringspot damage on grapefruits and oranges (Sakimura 1975). Described originally from Hawaii but presumably Southeast Asian in origin, it is known from Hawaii, Kauai, Maui and Oahu, is widely distributed among Caribbean islands and into Florida, but also known from Sumatra and Northeastern Australia.

########## DENDROTHRIPOIDES Bagnall

Of the five species in this genus, one is known from Zambia, two from the Philippines and the other two from Southeast Asia. The genus is highly recognizable, because of the large, broadly based microtrichia on the tergites (Fig. 56), and the transverse ridge across the head.

***Dendrothripoides innoxius* (Karny)**: One of the synonyms of this species is *ipomoeae*, a name that recognises that this thrips is often a pest on the leaves of *Ipomoea batatas*, the sweet potato. However, it has been found on several other plants, and possibly in association with leaf damage to lettuce (Zimmerman 1948). Recorded from Hawaii, Kauai, and Oahu, this species is widespread in Southeast Asia and across the Pacific from Australia to Panama, but is also recorded from Florida and Brazil.

########## DICHROMOTHRIPS Priesner

This genus includes 18 species from the Old World between Africa, Asia and Australia, and two of these are known from the Hawaiian Islands. Two further species, *semicognitus* and *phalaenopsidis*, were described by Sakimura (1955) from specimens taken in quarantine at Honolulu imported from the Philippines, and listed from Oahu in Nishida et al. (1992). But there is no record of either species living on the Hawaiian Islands. The members of this genus presumably all breed only on Orchidaceae, on which they sometimes cause economic damage, including *smithi* that is a pest on commercial Vanilla crops in India. As in *Taeniothrips* species, the 8-segmented antennae tend to have segments III and IV with the apex slender (Fig. 58), tergite VIII has a well developed regular comb of long microtrichia (Fig. 61), the tergites lack ctenidia, and there are no sternal discal setae. Males of species in *Dichromothrips* have prominent, paired pore plates on some sternites.

########### Key to species from Hawaiian Islands

**Table:** 

1	Fore wing banded, subapical area pale in contrast to dark apex and median area	***dendrobii***
–	Fore wing uniformly brown with base pale	**2**
2	Pronotum with no long posteroangular setae (Fig. 57)	***corbetti***
–	Pronotum with 1 pair of posteroangular setae more than twice as long as other posteromarginal setae	***smithi***

***Dichromothrips corbetti* (Priesner)**: Described from Malaysia, but widely reported around the world, particularly as damaging cultivated *Vanda* orchids, this species has characteristic transverse sculpture lines on the metanotum, and unlike other species in the genus lacks any long pronotal setae (Fig. 57). Common in Southeast Asia, and reported in greenhouses from Australia, it is known from Hawaii, Kauai, Maui and Oahu, as well as in orchid houses across the northern hemisphere.

***Dichromothrips dendrobii* Sakimura**: Despite the presence of long pronotal posteroangular setae, this species is similar in structure to *corbetti*, and is distinguished particularly by the banded fore wings. Presumably from the Philippines, it is reported from Oahu as damaging the leaves and flower buds of *Dendrobium superbum*.

***Dichromothrips smithi* (Zimmermann)**: This species has been taken several times by quarantine in California on orchids imported from Hawaii. It is also listed in an unpublished, updated list of Hawaiian insects prepared by Bernarr Kumashiro. Presumably established on the Hawaiian Islands, this thrips is known as a pest of Vanilla crops in India, and is recorded from Malaysia, Solomon Islands, Taiwan, and Japan.

########## ECHINOTHRIPS Moulton

A genus of seven species from various parts of North and South America, these thrips are dark brown, with the head and pronotum reticulate (Fig. 62), a row of long capitate setae on the fore wing first vein (Fig. 63) but with no setae on the second vein, tergal median setae close together (Fig. 64), tergite VIII with a complete comb, and the males with multiple small sternal pore plates (Mound and Marullo 1996). One species has become widespread around the world.

***Echinothrips americanus* Morgan**: Originally from the Eastern United States, this species is recorded from Hawaii and Oahu, and is now widespread as a greenhouse pest across the northern hemisphere (Vierbergen et al. 2006). It has also been found in Indonesia and Northern Australia (Mound et al. 2013). Originally associated with *Impatiens* species, in greenhouses it has been regarded as a pest of *Capsicum* as well as of ornamental plants.

########## FRANKLINIELLA Karny

This is one of the largest genera of Thysanoptera, with over 230 species, mostly from the Americas, but with a particularly large number of species from the Neotropics (Mound and Marullo 1996; Cavalleri and Mound 2012). Most of these species are flower-living, but a few live only on Poaceae, and several species are pests and tospovirus vectors. All of the species bear paired ctenidia on the posterior abdominal tergites, and on tergite VIII the ctenidia are anterolateral to the spiracles (Figs 71, 76). Ocellar setae pair I are present on the head in front of the first ocellus (Fig. 67), and the fore wings bear two continuous rows of setae. The pronotum usually bears four pairs of long setae, and there is a small pair of median setae between the major pair of median posteromarginal setae (Fig. 65). Males have pore plates on the sternites, and females of a very few species have pore plates on the second abdominal sternite.

########### Key to species from Hawaiian Islands

**Table:** 

1	Tergite VIII posterior margin with no comb, or comb represented only by 2–3 teeth laterally (Fig. 76)	**2**
–	Tergite VIII posterior margin with comb of long microtrichia, or of short microtrichia with broad triangular bases (Figs 72, 73), complete across entire margin or with small median gap equivalent to 2 or 3 missing microtrichia (Fig. 75)	**5**
2	Ocellar setae pair III arising within ocellar triangle (Fig. 67)	**3**
–	Ocellar setae pair III arising outside or on anterior margins of ocellar triangle (Figs 65, 66)	**4**
3	Head not produced in front of eyes; ocellar setae pair III arising between posterior ocelli, or on tangent between anterior margins of posterior ocelli (Fig. 67); tergite VIII posterior margin usually with no microtrichia (Fig. 76)	***schultzei***
–	Head slightly produced in front of eyes; ocellar setae pair III arising in front of tangent between anterior margins of posterior ocelli; tergite VIII posterior margin with a few microtrichia laterally	***bondari***
4	Pedicel of antennal segment III with sharp-edged ring, distal to which is a chalice-like collar (Fig. 69); tergite VIII posterior margin with a few small microtrichia laterally	***cephalica***
–	Pedicel of antennal segment III with margins parallel to softly rounded (Fig. 68); tergite VIII posterior margin with several broadly based scallops; sometimes micropterous	***fusca***
5	Ocellar setae pair III no longer than diameter of an ocellus, arising on outer margin of ocellar triangle (Fig. 66); pronotal anteromarginal setae short, no longer than width of antennal segment II; tergite VIII with posteromarginal comb teeth as long as S1 setae on tergite VI	***minuta***
–	Ocellar setae pair III more than 2.0 times as long as diameter of an ocellus; pronotal anteromarginal setae longer than width of antennal segment II; comb on VIII with teeth variable, but never as long as S1 setae on tergite VI	**6**
6	Pedicel of antennal segment III with sharp-edged ring (Fig. 70); comb on tergite VIII with widely spaced teeth	***invasor***
–	Pedicel of antennal segment III with margins parallel to softly rounded; comb on tergite VIII variously constructed	**7**
7	Fore wing brown with base sharply paler	**8**
–	Fore wing uniformly pale or weakly shaded	**9**
8	Metanotum with pair of campaniform sensilla present; tergite VIII comb teeth longer than their basal width, commonly with one or two absent medially (Fig. 75)	***insularis***
–	Metanotum with no campaniform sensilla; tergite VIII comb teeth shorter than their basal width, often irregular or bifurcate, and present across entire margin (Fig. 74)	***hemerocallis***
9	Body yellow with brown markings medially on tergites, or body brown	***occidentalis***
–	Body yellow, with no obvious darker markings	**10**
10	Sternite II usually with 1–3 discal setae medially (Fig. 77); tergite VIII comb with long regular teeth (Fig. 72); living on Poaceae leaves, particularly *Zea mays*	***williamsi***
–	Sternite II with no discal setae medially; tergite VIII comb with short irregular teeth (Fig. 73); living in flowers of *Crotalaria* species	***crotalariae***

***Frankliniella bondari* Hood**: Described from Brazil, and recorded from Cuba, Mexico, Florida and Georgia, this small yellow species was first taken on Molokai in 1990. It is now known from Hawaii, Molokai and Oahu, but although adults have been taken from *Polyanthus* flowers there is no information available on the range of plants on which it might breed.

***Frankliniella cephalica* (Crawford)**: This is one of the most common thrips in a wide range of flowers, particularly white flowers, in countries around the Caribbean, including Florida and Texas. It has been taken on Maui and Oahu, and is one of only two species from Hawaiian Islands with a sharp-edged disc on the pedicel of the third antennal segment. In contrast to *invasor*, tergite VIII posterior margin has only a few microtrichia and these are placed near the lateral margins.

***Frankliniella crotalariae* Mound & Marullo**: Previously known only from Costa Rica, this species is easily misidentified with *occidentalis*, but the abdomen is clear yellow, and the comb on tergite VIII is shorter and more irregular (Fig. 73). It has been taken in the flowers of *Crotalaria* species on Hawaii, and one sample collected by R.G. Hollingsworth at Pahala in August 2015 included both sexes and large numbers of larvae.

***Frankliniella fusca* (Hinds)**: Widespread from Canada to the Caribbean, including Mexico, Puerto Rico and Martinique and also introduced to The Netherlands, the tobacco thrips is a major pest in Georgia and Florida. It is polyphagous, and is a major vector of tospoviruses, causing problems on peanut and cotton crops. It is reported from Hawaii, but with no indication of being a pest locally. Adults of both sexes can be either fully winged (macropterous) or have wings shorter than the thorax width (micropterous).

***Frankliniella hemerocallis* Crawford**: The day-lily thrips appears to be monophagous on *Hemerocallis* leaves and flowers. Described originally from Wisconsin, it is widespread on its host in North America. It is also recorded from Bermuda, Costa Rica, Japan, and Hungary, as well as Oahu, but the country of origin is not clear. It is a large dark brown species with bicoloured fore wings.

***Frankliniella insularis* (Franklin)**: One of the most common thrips species of Central and South America, but also found in southern areas of USA, this species has been taken on Hawaii. Another large dark species, with bicoloured fore wings, it is commonly found between the petals of *Malvaviscus* flowers. However, it appears to breed particularly in the flowers of various cultivated Fabaceae, including *Cajanus* and *Pachyrhizus*, on which it can be a minor pest.

***Frankliniella invasor* Sakimura**: Although common in Hawaii, from where it was described originally, this species was presumably introduced from the Caribbean region. It is recorded from Hawaii, Kauai, Lanai, Maui, Molokai and Oahu, but is also known from Trinidad, St Vincent and Puerto Rico as well as Panama, Costa Rica, Guatemala and Mexico. It has been collected in the flowers of various plants, including *Gardenia*, *Leucaena*, and *Mangifera*. There are other closely similar yellow species with an angulate pedicel on the third antennal segment in flowers in the Caribbean region (Mound and Marullo 1996).

***Frankliniella minuta* (Moulton)**: Described from California originally, this species is widespread in the Western USA southwards through Central America to Peru, and also recorded from Lanai, Oahu and Midway Island. A polyphagous species, it is one of a group of *Frankliniella* species in which the setae on the head and pronotum are particularly short (Fig. 66), and in which species are usually found in the flowers of Asteraceae (Sakimura and O’Neill 1979).

***Frankliniella occidentalis* (Pergande)**: Western flower thrips is one of the most important pest insects around the world, and has been found on Hawaii, Kauai, Lanai, Maui and Oahu. Originally from the western USA, it is now found worldwide, and is the most important vector of the thrips-borne Tospovirus diseases (Riley et al. 2011). It is highly polyphagous, but at times it is a useful beneficial when it feeds on populations of leaf mites (Agrawal et al. 1999). The body colour is variable, being dark brown in cooler areas, but more commonly yellowish with brown markings on the tergites. Molecular data now indicates that this species may be a complex of non-interbreeding, genetically distinct, races that cannot be distinguished morphologically (Rugman-Jones et al. 2010)

***Frankliniella schultzei* (Trybom)**: The country of origin of this species, the tomato thrips, is not clear. It occurs as both a yellow and a brown form, and both forms are found worldwide. The extent of genetic variation and its significance among populations of *schultzei* remains to be studied effectively, and the colour forms possibly represent two or more different species. It has been claimed that only the brown form is a tospovirus vector, but populations of yellow individuals are important as virus vectors on vegetable crops in Northeastern Australia. The species is found particularly in the tropics and subtropics, and has been recorded from Hawaii, Kauai, Lanai, Niihau and Oahu. It is usually easily recognised because of the position of ocellar setae pair III, and the absence of a comb on tergite VIII.

***Frankliniella williamsi* Hood**: This species lives on various Poaceae, and is particularly associated with the leaves of *Zea mays*. Seedlings of field corn can be stunted or killed by this thrips, and seed production reduced by 20–40% (Mau and Gusukuma-Minoto 1999). It is also implicated in the mechanical transmission of maize chlorotic mottle virus of corn in Kauai and some states in mainland USA (Jiang et al. 1992). It is a large, clear yellow species, and is most easily recognised by the presence of discal setae on the second abdominal sternite.

########## KURTOMATHRIPS Moulton

Four species are listed in this genus (Borbon 2005), all from the Americas. Adults are usually wingless, with the body strongly sculptured, the head smaller than the trapezoidal pronotum, the antennae 8-segmented with no sense cone on segment III and one simple one on IV, and males with a transverse pore plate on sternites III–VII.

***Kurtomathrips morrilli* Moulton**: Body mainly yellow, with small brown markings, the pronotal posteroangular setae of this species are long and flattened (Fig. 78). It is widespread in Southwestern USA, but also found in Mexico and Jamaica, as well as Kauai, Maui and Oahu. Bailey (1957) reported it as damaging cotton and chrysanthemums in Arizona and California.

########## LIMOTHRIPS Haliday

Although eight species are listed in this European genus, three of these are known only from old descriptions from which they cannot be recognised (zur Strassen 2003). These thrips breed on the leaves and in the flowers of species of Poaceae, sometimes producing enormous populations, and some species have been widely distributed around the world. The terminal abdominal segments bear one or more distinctive pairs of very stout setae (Fig. 80).

***Limothrips cerealium* (Haliday)**: This is one of the most widespread species of thrips, found all across the temperate zone of the northern hemisphere, and also in Australia as well as Hawaii, Kauai and Oahu. Swarms of this thrips rising from cereal crops are common in Europe in warm stormy weather, giving the common name of “Thunder Flies” (Kirk 2004).

########## MICROCEPHALOTHRIPS Bagnall

Although similar to members of the genus *Thrips* in many characters, such as the presence and position of tergal ctenidia and the absence of pair I of the ocellar setae, the single species in this genus is remarkable for the presence of a group of setae on the prosternum (Fig. 81).

***Microcephalothrips abdominalis* (Crawford)**: As indicated by the name, the head of this species is unusually small, and the tergites and sternites bear a posteromarginal craspedum of pointed lobes (Fig. 82). It is widespread around the world, particularly in subtropical areas, and is common on various Pacific islands, including Hawaii, Kauai, Maui and Oahu. It breeds in the flowers of many different Asteraceae, including sunflowers.

########## NEURISOTHRIPS Sakimura

This is an endemic Hawaiian genus of which seven species have been described. These species share the following character states with *Frankliniella* species: antennae 8-segmented, ocellar setal pair I present, both fore wing veins with complete row of setae, metanotal median setae usually at anterior margin, sternites and pleurotergites without discal setae, male sternites III–VII with transverse pore plates. However, the two genera differ in that species of *Neurisothrips* lack tergal ctenidia, and their tergites and sternites bear a posteromarginal craspedum that varies in size among the species but is lobed between the posteromarginal setae. The following key is based on the holotypes of the species described by Moulton together with one of the original specimens of *multispinus*. These type specimens are all uncleared slide-mounts and unsuitable for the production of clear images. Also studied were over 50 slide-mounts prepared by the late Kanjyo Sakimura. From these slides it is possible to deduce that this endemic genus is far more species-rich than published information indicates. However, the available specimens are uncleared with many character states difficult to observe. To examine this unique endemic radiation, field studies are needed in the Hawaiian Islands to establish host associations and distributions for each species. Fresh material is needed for slide mounting and critical study of morphology, as well as DNA analyses.

########### Key to species

**Table:** 

1	Ocellar setae pair III shorter than distance between posterior ocelli; postocular setae pair IV scarcely longer than length of one ocellus (Fig. 84)	**2**
–	Ocellar setae pair III and postocular setae pair IV much longer (Figs 84, 85)	**3**
2	Fore wing with dark median band and dark apex; body, legs and all antennal segments brown; tergites laterally with group of less than 5 setae	***fasciatus***
–	Fore wing pale without dark areas; body, legs and antennal segments I–IV also base of V and VI yellow (Fig. 91); tergites laterally with group of about 7 setae	***dubautiae***
3	Body largely yellow	**4**
–	Body brown to dark brown	**5**
4	Tergites with transverse row of 3 or 4 dark setae laterally (Fig. 86); antennal segments I–II yellow, III–VII dark brown; metanotal median setae arise almost medially, well behind anterior margin	***carteri***
–	Tergites laterally with irregular group of about 7 setae; antennal segment I white, II light brown, and III yellow in basal quarter; metanotal median setae arise at anterior margin	***williamsi***
5	Antennal segment III 75 microns long (60 microns in male), slender with constricted apex (Fig. 89)	***antennatus***
–	Antennal segment III no more than 50 microns long, apex usually less constricted (Fig. 90)	**6**
6	Postocular setae pair IV 75 microns long	***fullawayi***
–	Postocular setae pair IV 50 microns long	***multispinus***

***Neurisothrips antennatus* (Moulton)**: Although described originally from a single male taken on Oahu, this species has been recorded subsequently from Hawaii, Kauai, Lanai, Maui, and Molokai. It has been collected from various plants, including *Astelia*, *Menziesiana*, *Broussaisia*, and *Metrosideros*.

***Neurisothrips carteri* (Moulton)**: Described from a single female taken in a wind trap on Oahu, the arrangement of the setae on the lateral areas of the abdominal tergites seems to be unique (Fig. 86). Moreover, the metanotal median setae arise well behind the anterior margin of that sclerite. The species has been recorded from Hawaii, Molokai, and Oahu.

***Neurisothrips dubautiae* (Moulton)**: The type series of this yellow species comprised seven females and 10 males taken on Oahu from *Dubautia*, one of the native Asteraceae. Ocellar setae pair III are only 25 microns long, and postocular setae pair IV are about the same length.

***Neurisothrips fasciatus* (Moulton)**: Described from a single female taken in a wind trap on Oahu, this species is unique in the genus in having banded fore wings. However it is similar to *dubautiae* in having unusually short setae on the head, with ocellar setae pair III 25 microns long, and postocular setae pair IV scarcely 15 microns. The abdomen of the holotype is shrunken, thus obscuring details, but there seem to be fewer setae laterally on the tergites than in the other members of this genus.

***Neurisothrips fullawayi* (Moulton)**: This brown species appears to be closely similar to *multispinus*, and longer series of specimens are needed to provide a clearer distinction between them. Described from four females and one male, it has been recorded from Hawaii, Kauai, Molokai, and Oahu from various plants including *Broussaisia*, *Broussonetia* and *Pipturus*.

***Neurisothrips multispinus* (Bagnall)**: Recorded from Hawaii, Kauai, Maui, and Oahu, this is possibly the most common species in this genus, and has been taken from the flowers of various plants including *Ipomoea* sp. and *Styphelia tameiameiae*. A dark brown species, it is not currently distinguished satisfactorily from *fullawayi*.

***Neurisothrips williamsi* (Moulton)**: A yellow species with the tergal antecostal ridges and the major setae dark, this species was described from three females. Antennal segments III and IV have their apices narrowed, and there is a group of well-developed setae laterally on the tergites as in *multispinus* (Fig. 87). It has been recorded from Hawaii, Kauai and Oahu, from the flowers of various plants including *Acacia koa*, *Metrosideros*, and *Myoporum sandwicense*.

########## ORGANOTHRIPS Hood

Three species are recognised in this genus, although the distinction between *Organothrips bianchi* from the Pacific and *Organothrips indicus* from Asia is not entirely clear (Mound 2000). The genus is recognised from the curious fringed spur at the inner apex of the fore tibia (Fig. 93), and the prolongation of the head in front of the eyes (Fig. 92). These thrips all live on aquatic plants, apparently breeding within mucous on the plant stems below water-level.

***Organothrips bianchii* Hood**: Described from Oahu on *Colocasia esculenta*, this species is also known from Kauai, Lanai, Maui, Molokai and Oahu, as well as the Palau Islands and Samoa. The fore wing is unusual in shape, curving forwards with the apex slightly rounded.

########## PEZOTHRIPS Karny

This genus is possibly polyphyletic. Nine of the 10 included species are from the Palaearctic region, between Europe and Tibet. In contrast, *kellyanus* is considered Australian in origin but has been introduced to southern Europe. At one time this genus was confused with *Megalurothrips*, but all the species in that genus breed in the flowers of Fabaceae.

***Pezothrips kellyanus* (Bagnall)**: Kelly’s citrus thrips is considered to be originally from eastern Australia, where it is common in various scented, white flowers. However, this citrus pest has been introduced to southern Europe, and is common in New Zealand and New Caledonia. It was intercepted by quarantine in California from Hawaii in 1998, recorded in a pre-departure agricultural inspection at Kona in 2000, and in 2014 was collected from flowers on Hawaii where it is clearly established. The adults have antennal segment III sharply bicoloured, with the constricted apex white in contrast to the dark brown of the rest of this segment (Fig. 98). Males have longer antennae, and there is a series of 20 or more small pore plates on each sternite.

########## PLESIOTHRIPS Hood

This is a New World genus with 19 species listed from various parts of North and South America. Females of these species all have the ovipositor exceptionally weak and lacking strong serrations. Moreover, the males have antennae very different from those of females, with antennal segment III very short but segments IV to VI elongate. All of these species live on Poaceae, but there are no studies on their biology.

***Plesiothrips perplexus* (Beach)**: This is the most widespread member of the genus. It is known widely across the Americas, and is common among the Caribbean and Pacific islands, as well as the warmer parts of Australia and New Zealand. It is recorded from Hawaii, Kauai, Lanai, Molokai and Oahu, where it has been taken from many different grass species, including sugar cane. Like many common thrips species, adults vary in colour, with the abdomen commonly paler than the brown head and thorax.

########## PROJECTOTHRIPS Moulton

The nine species listed in this genus apparently all live in the flowers of *Pandanus* species, the screw pines, between India, northern and eastern Australia, and the Pacific islands. The antennae are characteristic, with segment VIII long and spindle-shaped (Fig. 110).

***Projectothrips trespinus* (Moulton)**: Described from Oahu, but also recorded from Hawaii, Kauai and Maui, this species is possibly endemic to the Hawaiian Islands. Adults have been collected from various plants, but the species presumably breeds only in the flowers of *Pandanus*.

########## PSEUDANAPHOTHRIPS Karny

Eight of the nine species listed in this genus are from Australia, with one described from Taiwan. However, one of the Australian species is introduced to the Hawaiian Islands along with its host plant. The species of *Pseudanaphothrips* are similar to *Frankliniella* species in the following characters: both fore wing veins with complete setal rows, antennae 8-segmented, ocellar setae pair I present in front of the first ocellus. However, *Pseudanaphothrips* species lack the pair of small setae medially on the pronotal posterior margin, and tergal ctenidia are absent or very weak.

***Pseudanaphothrips araucariae* Mound & Palmer**: This thrips lives in the male cones of *Araucaria bidwilli* in Australia, but has been found in large numbers on Oahu and Hawaii in the male cones of *Araucaria heterophylla*, the Norfolk Island pine. It has also been seen from Tahiti. There is a pair of well-developed ctenidia on tergite VIII, but the preceeding tergites bear a scattering of microtrichia on various sculpture lines.

########## PSYDROTHRIPS Palmer & Mound

Two species are listed in this Neotropical genus. The first was described from a greenhouse in England but subsequently recorded from Mexico, and the second was described from Oahu. It is one of the few Thripidae with 9-segmented antennae (Fig. 109), the sternites bear discal setae, and tergite VIII has a long posteromarginal comb.

***Psydrothrips luteolus* Nakahara & Tsuda**: Described from Oahu, this species is also known from Florida. It causes damage and malformation to the terminals of Araceae plants, and Edwards (1995) recorded considerable feeding damage to unfurled leaves of commercial *Spathiphyllum*, leading to longitudinal scars on open leaves.

########## RHAMPHOTHRIPS Karny

There are 17 species listed in this genus, all from the Old World tropics. The genus is characterised by the close proximity of posteromarginal setal pairs I and II medially on sternite VII of females (Fig. 104). Moreover, the head is small in comparison to the pronotum, and the mouth cone unusually long. The tergites and sternites have a posteromarginal craspedum.

***Rhamphothrips pandens* Sakimura**: Although described from Hawaii, the country of origin of this small yellow species remains unclear. It has been recorded widely across the Pacific, from Northern Australia and New Caledonia, through Polynesia and Kiribati to Florida, Jamaica and other Caribbean territories (Mound and Tree 2011). The species appears to feed on the leaves of a wide range of plants in various families.

########## SCIOTHRIPS Bhatti

A single species is placed in this Asian genus. It has the head prolonged in front of the eyes (Fig. 106), and tergite VIII bears a long posteromarginal comb of microtrichia. Structurally similar to *Taeniothrips*, the genus is distinguished in having fewer setae on the fore wing second vein.

***Sciothrips cardamomi* (Ramakrishna)**: Apparently specific to the flowers of certain Zingiberaceae, including cardamom, of which it is considered a pest in India. It has also been recorded from such plants in Hawaii and Costa Rica (Mound and Marullo 1996).

########## SCIRTOTHRIPS Shull

This genus includes about 110 species from various warmer parts of the world. Both sexes have closely spaced rows of fine microtrichia on the lateral thirds of the abdominal tergites (Fig. 103), the pronotum bears many closely spaced transverse striae, and antennal segment II has the inner major seta larger than the outer seta. Females lack campaniform sensilla on tergite IX, and males of some species have paired lateral drepanae on tergite IX. These are leaf-feeding thrips, usually associated with young leaves and developing fruits rather than flowers, and several species are important pests.

########### Key to species from Hawaiian Islands

**Table:** 

1	Head with ocellar setae pair III arising between the posterior ocelli (Fig. 101); fore wings with posterior fringe cilia straight	**2**
–	Head with ocellar setae pair III arising within ocellar triangle, between anterior and posterior ocelli (Fig. 100); fore wings with posterior fringe cilia wavy	**3**
2	Abdominal sternites with rows of microtrichia extending across median area (Fig. 102); tergites with brown area on median third	***dorsalis***
–	Abdominal sternites with microtrichia restricted to lateral areas; tergites pale medially but antecostal ridges shaded	***inermis***
3	Pronotum with longest posteroangular setae about 3 times as long as pronotal discal setae; tergites with no dark transverse marking; many closely spaced transverse lines between posterior ocelli	***citri***
–	Pronotum with longest posteroangular setae less than 2 times as long as discal setae; tergites with dark transverse antecostal ridge; few widely spaced lines between posterior ocelli	***perseae***

***Scirtothrips citri* (Moulton)**: The Californian citrus thrips is widespread in Arizona, California, New Mexico, Texas and Mexico. Moreover, populations that may represent this species can be found across the southern states to Florida. A further complication is that some other *Scirtothrips* species described from California are very similar to *citri* and may not be biologically distinct (Hoddle et al. 2012). The major native host plant in California is considered to be a species of *Rhus*, but *citri* is a polyphagous insect, and on Maui has been found on *Citrus* and *Mangifera*.

***Scirtothrips dorsalis* Bagnall**: This Asian species is widespread from India to Japan and Australia, and has been introduced to Florida and Israel. Highly polyphagous, it is a serious pest on a range of crops across these countries. However, there are problems concerning the identity of some populations, both from morphological data (Mound and Stiller 2011), and from molecular data (Hoddle et al. 2008b). It has been taken on Maui and Oahu from various plants, but the pest status of these populations remains unclear. It is one of a small number of species in the genus *Scirtothrips* in which rows of microtrichia extend medially fully across the abdominal sternites.

***Scirtothrips inermis* Priesner**: Described from the Canary Islands, this species has been reported from various parts of the world, including Europe, Australia, California and Maui. However, it has usually been collected in low numbers, with no evidence of economic damage to any plants. On Norfolk Island it was found to be quite common on the young leaves of several fruit tree crops, but again without evidence of any damage (Mound and Wells 2015).

***Scirtothrips perseae* Nakahara**: Described from California as a pest on avocado leaves and young fruits, five further synonyms of this species are known from Mexico (Hoddle et al. 2008a). There is a record of this species from Maui without further data in an unpublished, updated list of Hawaiian insects provided by Bernarr Kumashiro during 2015.

########## SCOLOTHRIPS Hinds

The 14 species included in this genus are well-known as predators of spider mites on a wide range of plants, and are sometimes marketed as biocontrol agents. The genus was erected for *sexmaculatus* Pergande, a species described from California but known widely across the USA. The name *sexmaculatus* has also been used for populations in other parts of the world, but these generally are misidentifications of different species that are restricted to the Old World (Mound 2011a). Members of the genus are easily recognized from the presence of six pairs of unusually long setae on the pronotum (Fig. 105), and they usually have the body largely pale, and the fore wings with small dark transverse markings.

########### Key to species from Hawaiian Islands

**Table:** 

1	Pronotum with pair of posteromedian discal setae (Fig. 105); fore wing with subbasal brown area not reaching anterior margin, subapical brown area usually larger than subbasal area	***pallidus***
–	Pronotum without paired posteromedian discal setae; fore wing with subbasal and subapical brown areas longer than wide	***takahashii***

***Scolothrips pallidus* (Beach)**: Described from Iowa, and recorded from Oahu as well as from various localities between Canada and Mexico, this species is possibly no more than a pale form of *Scolothrips sexmaculatus*, the six-spotted thrips Mound (2011a).

***Scolothrips takahasii* Priesner**: Under the name *priesneri* Sakimura, this species has been recorded from Hawaii, Kauai, Maui, Molokai and Oahu. However, it was first described from Taiwan, and the Hawaiian species was recognized as a synonym by Mound (2011a).

########## STENCHAETOTHRIPS Bagnall

This genus is closely related to the genus *Thrips*, and all 38 described species apparently breed only on species of Poaceae. These thrips share all the character states of genus *Thrips*, but the pair of setae lateral to the fore ocellus on the head (setal pair II) is longer than setal pair III that is associated with the ocellar triangle. Most of the species are from Southeast Asia, although the one first described was from Sudan.

***Stenchaetothrips minutus* (van Deventer)**: Described from Java originally, synonyms of this species were described from India, Brazil and Hawaii. It has been found living on various Poaceae, including sugar cane and maize, and is recorded from Hawaii, Kauai, Molokai and Oahu. Unlike many species in this genus, the fore wings are brown with only the basal quarter paler.

########## TAENIOTHRIPS Amyot & Serville

This genus includes 30 species from the Holarctic region and Southeast Asia (Mound et al. 2012), together with 20 species known only from fossils. These flower-living species are similar to members of the genus *Thrips* in lacking a pair of setae in front of the fore ocellus, but the two genera are unrelated in view of the absence of any tergal ctenidia on *Taeniothrips* species.

***Taeniothrips eucharii* (Whetzel)**: First described from Bermuda, with two synonyms from Taiwan and Japan, this thrips is widespread in association with various Amaryllidaceae, including *Hymenocallis*, *Eucharis*, *Lycoris*, *Narcissus* and *Zephyranthes*, and is common on *Liriope* (Liliaceae) in Japan (Mound and Tree 2009). It has presumably been moved around the world by the horticultural trade in bulbs, presumably originally from Southeast Asia, and is recorded from Hawaii, Molokai and Oahu. It is a typical member of the genus *Taeniothrips*, with long ocellar setae pair III arising within the triangle (Fig. 107), and a long regular comb of microtrichia on the eighth abdominal tergite.

########## TENOTHRIPS Bhatti

Nineteen species are listed in this genus, and these differ from *Taeniothrips* species in having a pair of setae in front of the first ocellus, that is, ocellar setal pair I is present. The genus appears to comprise two major groups. One group of species is from the Old World, mainly Europe, but the second group is known only from California and is referred to by Hoddle et al. (2012) under the generic name *Ewartithrips*.

***Tenothrips frici* (Uzel)**: Described from Europe, this small brown species is widespread around the temperate parts of the world in the yellow flowers of weedy Asteraceae, particularly *Hypochoeris* species. It is established in higher elevations of the Hawaiian Islands where the temperature is cooler, such as Kula area in Maui and the saddle road area in Hawaii. The sculpture of the posterior half of the metanotum is characteristic of this species (Fig. 108).

########## THRIPS Linnaeus

More than 290 species are listed in this genus from around the world, although none of these is native to the Neotropics. These species all lack pair I of the ocellar setae, and they all have paired ctenidia laterally on the abdominal tergites, the pair on tergite VIII arising posteromesad of the spiracles (Fig. 15). Other characters, such as the number of fore wing veinal setae, and discal setae on the sternites and pleurotergites are variable among species. Nakahara (1994) provided keys to the North American species of this genus, and Mound and Masumoto (2005) keys to the Australasian species.

In addition to the 13 species in this genus dealt with below, *Thrips trehernei* was recorded by Zimmerman (1948) collected from a pineapple at Honolulu in 1930. The original specimen is correctly identified, and is deposited in the California Academy of Sciences collection. However, in the absence of any further record from the Hawaiian Islands this European species is not considered further here. It breeds specifically in certain yellow-flowered Asteraceae, particularly *Taraxacum* species.

########### Key to species from Hawaiian Islands

**Table:** 

1	Abdominal sternites III–VI with no discal setae	**2**
–	Abdominal sternites III–VI with at least one pair of discal setae present (Figs 128–130)	**4**
2	Body clear yellow; metanotum with pair of campaniform sensilla present (Fig. 114); tergite II lateral margin with 4 setae	***palmi***
–	Body with at least some darker markings; metanotum without paired campaniform sensilla; tergite II with 3 lateral marginal setae	**3**
3	Ocellar pigment grey; abdominal pleurotergites, also lateral areas of tergites, with closely spaced rows of ciliate microtrichia (Fig. 125); fore wing first vein with 4 or more setae on distal half (rarely only 3); compound eyes without pigmented facets; tergites with median setal pair short	***tabaci***
–	Ocellar pigment red; pleurotergites and tergites without ciliate microtrichia; commonly micropterous, macropterae with 3 setae on first vein distal half; compound eyes with 5 pigmented facets; tergites with median setal pair 0.5 as long as the tergite (Fig. 126)	***nigropilosus***
4	Sternite VII with no discal setae (Fig. 129)	**5**
–	Sternite VII with several discal setae	**6**
5	Sternites III–VI with 5–15 discal setae (Fig. 129); fore wing brown with base pale; metanotum reticulate with few or no markings inside the reticles	***parvispinus***
–	Sternites III–VI with 0–6 discal setae (Fig. 128); fore wing uniformly grayish-brown; metanotum reticulate with many markings inside the reticles (Fig. 113)	***orientalis***
6	Metanotum with conspicuous reticulation (Figs 112, 116)	**7**
–	Metanotum with longitudinal sculpture lines at least on posterior half (Figs 115, 117)	**10**
7	Fore wing first vein with complete setal row; fore wing clavus with 6 marginal setae	***australis***
–	Fore wing first vein with long interruption in setal row; clavus with 5 marginal setae	**8**
8	Antennae 7-segmented; pleurotergites each with 2 or 3 discal setae; sternites IV–V with 15-25 discal setae	***imaginis***
–	Antennae 8-segmented; pleurotergites without discal setae; sternites IV–V with less than 15 discal setae	**9**
9	Metanotal median setae arising behind anterior margin (Fig. 116)	***simplex***
–	Metanotal median setae arising at anterior margin	***macullicollis***
10	Abdominal pleurotergites with discal setae present	***alliorum***
–	Abdominal pleurotergites with no discal setae	**11**
11	Tergite VIII posterior margin without microtrichial comb (Fig. 124)	***vitticornis***
–	Tergite VIII posterior margin with complete comb of microtrichia (Fig. 127)	**12**
12	Fore wing clavus with terminal seta longer than subterminal seta (Fig. 123); mesonotum with sculpture lines around anterior campaniform sensilla (Fig. 121); antenna 7 or 8 segmented	***hawaiiensis***
–	Fore wing clavus with terminal seta shorter than subterminal seta (Fig. 122); mesonotum with no sculpture lines around anterior campaniform sensilla (Fig. 120); antenna 7-segmented	***florum***

***Thrips alliorum* (Priesner)**: Described from Taiwan, with one synonymous species described from Hawaii, *alliorum* is known from Japan, Korea and China living on the leaves of onion plants. It is also recorded from Hawaii, Kauai, Lanai, Maui, Molokai and Oahu. In contrast to the onion thrips, *Thrips tabaci*, it is a dark brown species with red ocellar pigments, the pleurotergites and sternites bear discal setae, and tergite VIII has a very weak posteromarginal comb.

***Thrips australis* (Bagnall)**: Originally from Australia, the gum tree flower thrips is now found in almost every country where *Eucalyptus* trees are planted, and it is reported from Hawaii, Kauai, Lanai, Maui, Molokai and Oahu. Adults may alight on many different plants, but this thrips is largely restricted for breeding to the flowers of *Eucalyptus* species and a few related Myrtaceae. Antennal segment VI is distinctively bullet-shaped (Fig. 119), the chaetotaxy of the fore wing and clavus is unusual, and the metanotum has equiangular reticulation with no internal markings and the median setae distant from the anterior margin (Fig. 112).

***Thrips florum* Schmutz**: Recorded from Hawaii and Oahu, this common Asian species is now found widely from Sri Lanka to northern Australia and across the Pacific. It is also recorded from Florida, and in parts of the Caribbean including the Bahamas, Puerto Rico, Costa Rica, Dominican Republic and Guatemala. It is a polyphagous flower-living species, but seems to be of limited economic importance. It is further discussed below under *hawaiiensis*.

***Thrips hawaiiensis* (Morgan)**: Recorded from Hawaii, Kauai, Lanai, Maui, Molokai, Midway, Niihau and Oahu, this is the most common flower thrips in the Hawaiian Islands. In many tropical parts of the world it is found in association with *florum*, but although both species are variable they can be distinguished on the characters indicated in the key above. The variation in size and colour of adult females was studied by Palmer and Wetton (1987), but the most common form of *hawaiiensis* seen from Oahu in recent years is larger and darker than forms more commonly found across Asia. Both this species and *florum* have been described under many different names.

***Thrips imaginis* Bagnall**: The Australian plague thrips was recorded from *Styphelia tameiameiae* flowers at Haleakala National Park, Hawaii in July, 2003 (Annual Report Hawaii Dept. Agriculture, 2006). An internal report at the Systematic Entomology Laboratory, USDA, Beltsville, indicated that three females from this sample were studied, but in 2015 these could not be found. This thrips has not previously been recorded east of New Caledonia, has never been reported by quarantine in California, and the record from Hawaii requires further confirmation. It is a polyphagous and abundant species in southern Australia (Mound and Masumoto 2005).

***Thrips maculicollis* (Hood)**: Described from northern Australia, and otherwise known only from New Caledonia (Mound and Masumoto 2005), this species has been intercepted several times by quarantine officials in California on leis originating in Hawaii and made from the flowers of *Fagraea berteriana*. According to Bernarr Kumashiro (in litt. 2015) this thrips has probably been established on Oahu and Hawaii for some considerable time.

***Thrips nigropilosus* Uzel**: This is a polyphagous, leaf-feeding, yellow species that is known to cause damage to various Asteraceae, including lettuce crops. Originally from Europe, it is widespread around the world, and reported from Hawaii, Kauai, Maui and Oahu. It is unusual within genus *Thrips* for being wing polymorphic, and even fully winged females have the median pair of setae on the tergites unusually long and almost half the length of their tergite (Fig. 126).

***Thrips orientalis* (Bagnall)**: Commonly associated with the white, scented flowers of *Gardenia* and *Jasminum* species, this thrips is recorded from Hawaii and Oahu. It is widespread from India to northern Australia and across the Pacific, but has also been recorded from Tanzania, the Virgin Islands, and Florida. It is sometimes a confusing species to identify, because the number, even the presence, of discal setae on the sternites is so variable between individuals. It is necessary to examine each sternite in order to check this character when identifying a specimen with a reticulate metanotum (Fig. 128).

***Thrips palmi* Karny**: This is considered a major pest on the leaves of various vegetable crops across much of Asia, from India to Japan and northern Australia, and is known as a vector of tospoviruses (Murai 2002). It is recorded from Hawaii, Kauai, Maui, Molokai and Oahu, and has been introduced widely to warmer parts of the Americas and also to some African territories. It is a small yellow species that is similar in structure to the common Eurasian species *flavus*, but has ocellar setae pair III wider apart and arising just outside the ocellar triangle.

***Thrips parvispinus* (Karny)**: Described from Thailand, and widespread in Southeast Asia to northern Australia, this species was found in 2006 damaging the young leaves of papaya at Puna and Hilo Hawaii. As indicated in the key above, it is similar in structure to *orientalis*, but seems to feed more frequently on leaves rather than flowers.

***Thrips simplex* (Morison)**: The gladiolus thrips is probably found wherever these plants are cultivated. It feeds on the leaves and flowers, and the feeding damage is particularly obvious on red flowers as pale linear markings. A dark species, it is recorded from Hawaii, Kauai, Maui, and Oahu. It is unusual in having about seven setae on the distal half of the fore wing first vein, and in having markings within the reticles on the metanotum (Fig. 116).

***Thrips tabaci* Lindeman**: The onion thrips is a ubiquitous, polyphagous species, although it is not usually found in the wet tropics. It is well known as a pest of onion and garlic plants, and is also a tospovirus vector, and it is recorded from Hawaii, Kauai, Kure, Lanai, Maui, Midway, Niihau, and Oahu. Adults are unusual with the genus Thrips for having grey, instead of red or orange, ocellar pigment, and a useful diagnostic character is the presence of closely spaced the rows of microtrichia on the pleurotergites (Fig. 125). Males of *tabaci* are rarely found outside the eastern Mediterranean countries where the host genus *Allium* is native.

***Thrips vitticornis* (Karny)**: Described from Thailand, with one synonymous species from Guam, this thrips is widespread from India to the South Pacific islands and northern Australia. A dark species, it is recorded from Hawaii, Kauai, Maui and Oahu, and is recognizable by the closely striate sculpture on the metanotum (Fig. 115). It appears to be associated with the flowers of certain Fabaceae, including *Canavallia* and *Calopogonium* species.

########## TRICHROMOTHRIPS Priesner

Currently there are 34 species listed in this genus, and Bhatti (2000) provided a full account of the 27 species that were known at that time. Many of these species were treated originally as *Dorcadothrips* species, a genus now considered a synonym. Most of the species are from Southeast Asia. Adult females lack ocellar setae pair I in front of the fore ocellus (Fig. 132), tergite VIII lacks a posteromarginal comb, and tergite X has no longitudinal split. The males commonly have a pair of drepanae on tergite IX, and sternites with multiple small pore plates.

########### Key to species from Hawaiian Islands

**Table:** 

1	Abdominal sternites with discal setae; tergal antecostal ridge pale brown	***cyperaceae***
–	Abdominal sternites without discal setae; tergites uniformly pale yellow	**2**
2	Head brown between eyes but pale yellow posterior to eyes; female sternites V–VI with submarginal brown area bearing callosity; male sternites III–VII with 5 pore plates, median one transverse	***xanthius***
–	Head pale yellow between eyes with brown band posterior to eyes; female sternites without submarginal brown callosity; male sternites III–VII with 6 pore plates, the 2 median ones oval	***oahuensis***

***Trichromothrips cyperaceae* (Bianchi)**: Described originally from Hawaii, and also reported from Maui and Oahu, this species has been found in southern California and Bermuda. Apparently specific to *Cyperus rotundus*, it is probably introduced from somewhere in Southeast Asia.

***Trichromothrips oahuensis* Nakahara**: Described from a few specimens collected from *Ocimum basilicum* on Oahu, the only other published record of this species is from Rurutu on the Austral Islands (Hoddle et al. 2008c). It is probably native to southeast Asia.

***Trichromothrips xanthius* (Williams)**: This species is sometimes a pest of orchids, including *Cattleya* and *Cypripedium* species, but has also been reported from unrelated greenhouse plants including *Asystasia* and *Chrysanthemum*. Described from Trinidad but probably originally from Southeast Asia, it has been reported in greenhouses widely across North America, Europe, Japan, and Australia.
